# Synthesis, Structural and Behavioral Studies of Indole Derivatives D2AAK5, D2AAK6 and D2AAK7 as Serotonin 5-HT_1A_ and 5-HT_2A_ Receptor Ligands

**DOI:** 10.3390/molecules28010383

**Published:** 2023-01-02

**Authors:** Agnieszka A. Kaczor, Ewa Kędzierska, Tomasz M. Wróbel, Angelika Grudzińska, Angelika Pawlak, Tuomo Laitinen, Agata Bartyzel

**Affiliations:** 1 Department of Synthesis and Chemical Technology of Pharmaceutical Substances with Computer Modeling Laboratory, Faculty of Pharmacy, Medical University of Lublin, 4A Chodźki St., PL-20093 Lublin, Poland; 2 School of Pharmacy, University of Eastern Finland, FI-70211 Kuopio, Finland; 3 Department of Pharmacology and Pharmacodynamics, Faculty of Pharmacy, Medical University of Lublin, 4A Chodźki St., PL-20093 Lublin, Poland; 4 Department of General and Coordination Chemistry and Crystallography, Institute of Chemical Sciences, Faculty of Chemistry, Maria Curie-Skłodowska University, M. Curie-Skłodowskiej Sq. 2, PL-20031 Lublin, Poland

**Keywords:** anxiety, depression, drug design, GPCRs, indole derivatives, molecular modeling, X-ray studies

## Abstract

Serotonin receptors are involved in a number of physiological functions and regulate aggression, anxiety, appetite, cognition, learning, memory, mood, nausea, sleep, and thermoregulation. Here we report synthesis and detailed structural and behavioral studies of three indole derivatives: D2AAK5, D2AAK6, and D2AAK7 as serotonin 5-HT_1A_ and 5-HT_2A_ receptor ligands. X-ray studies revealed that the D2AAK5 compound crystallizes in centrosymmetric triclinic space group with one molecule in the asymmetric unit. The main interaction between the ligands and the receptors is the salt bridge between the protonatable nitrogen atom of the ligands and the conserved Asp (3.32) of the receptors. The complexes were stable in the molecular dynamic simulations. MD revealed that the studied ligands are relatively stable in their binding sites, with the exception of D2AAK7 in the serotonin 5-HT_1A_ receptor. D2AAK7 exerts anxiolytic activity in the EPM test, while D2AAK5 has a beneficial effect on the memory processes in the PA test.

## 1. Introduction

G protein-coupled receptors (GPCRs) are the largest family of targets for approved drugs [1]. GPCRs serotonin receptors play an important role in the physiology and pathophysiology of the central nervous system (CNS). A neurotransmitter serotonin modulates neural activity and a number of neuropsychological processes, and drugs that target serotonin receptors are applied to treat a wide range of psychiatric and neurological disorders [2]. In particular, serotonin 5-HT_1A_ and 5-HT_2A_ receptors are involved in regulation of mood, cognition, and memory and are targeted by drugs used to treat depression, schizophrenia, and anxiety. Serotonin 5-HT_1A_ receptors agonists are effective in depression while agonistic or partial agonistic activity at this receptor is an important feature of many antipsychotics. Serotonin 5-HT_2A_ receptor antagonists display antipsychotic and antidepressant activity, while agonists have procognitive and hallucinogenic activity [3] and may be also used to treat treatment-resistant depression as in case of psychedelics [4].

**Figure 1:**
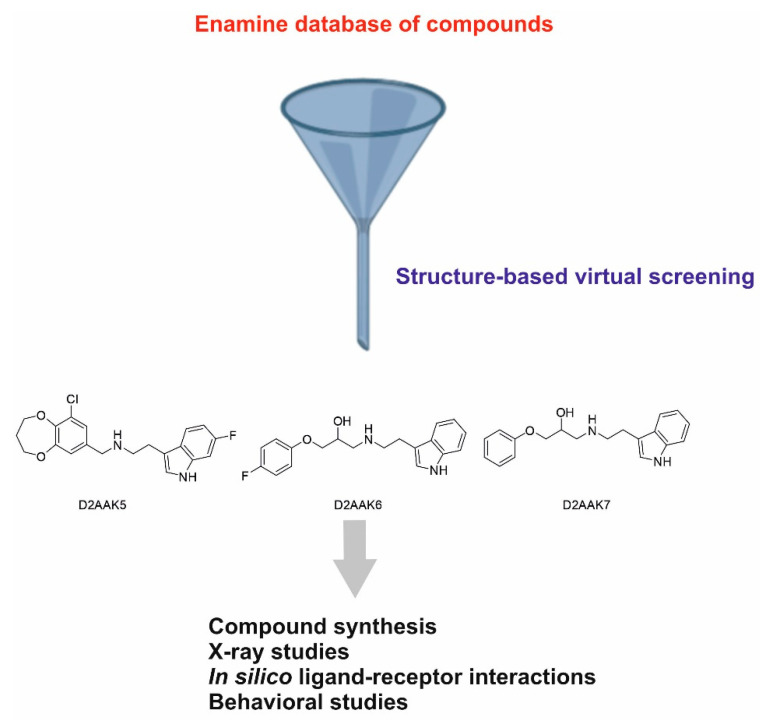
Rationale of the study and structural formulas of the studied compounds.

Searching for novel CNS active drugs, we performed structure-based virtual screening [5] and identified a number of potential antipsychotics acting through dopamine and serotonin receptors. D2AAK1 [6] and its derivatives [7,8], D2AAK3 [9], and D2AAK4 [10] display antipsychotic activity in animal models as they decrease amphetamine-induced hyperactivity. Moreover, all these compounds display pro-cognitive properties and some of them have anxiolytic and antidepressant activity. In this structure-based virtual screening experiment we also discovered D2AAK5, D2AAK6 and D2AAK7 (Figure 1), which do not possess a considerable affinity to dopamine receptors but display affinity to serotonin 5-HT_1A_ and 5-HT_2A_ receptors. The common feature of these three compounds is the indole scaffold in their structure which is one of the key privileged scaffolds in drug design and discovery [11].

Here we present synthesis and detailed structural and behavioral studies of D2AAK5, D2AAK6, and D2AAK7, which may facilitate successful design of derivatives of these compounds for potential treatment of depression, memory impairment, and anxiety.

## 2. Results

### 2.1. Chemistry

**Scheme 1:**
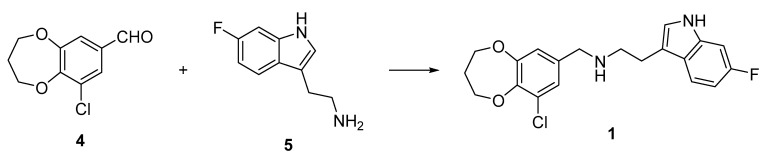
Synthesis of **1** (D2AAK5). Reagents and conditions: NaBH_4_, Na_2_SO_4_, MeOH, 12 h, room temperature.

Target compound **1** (D2AAK5) was synthesized in the form of hydrochloride via reductive amination between aldehyde **4** and 6-fluorotryptamine **5** (Scheme 1). The respective intermediates **4** and **5** were obtained according to the published methods [12,13,14,15,16]. Briefly, commercially available vanillin was chlorinated and demethylated to afford chlorodihydroxybenzaldehyde into which dioxepine ring was introduced by the action of dibromopropane, thus producing aldehyde **4**. Tryptamine **5** was obtained in three step sequence from commercially available 6-fluoroindole which was subjected to formylation via Vilsmeier-Haack reaction, converted into nitrovinyl derivative, and ultimately reduced with LiAlH_4_.

**Scheme 2:**
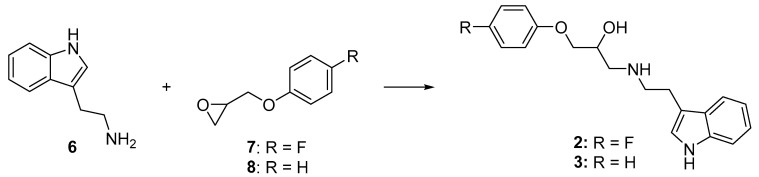
Synthesis of **2** (D2AAK6) and **3** (D2AAK7). Reagents and conditions: iPrOH, room temperature or reflux, 1 h or overnight.

Desired compounds **2** (DAAK6) and **3** (D2AAK7) were obtained as racemic mixtures in the form of hydrochlorides in one step from commercially available tryptamine **6** and also commercially available glycidol derivatives **7** and **8,** respectively (Scheme 2).

### 2.2. X-ray Studies of D2AAK5

**Figure 2:**
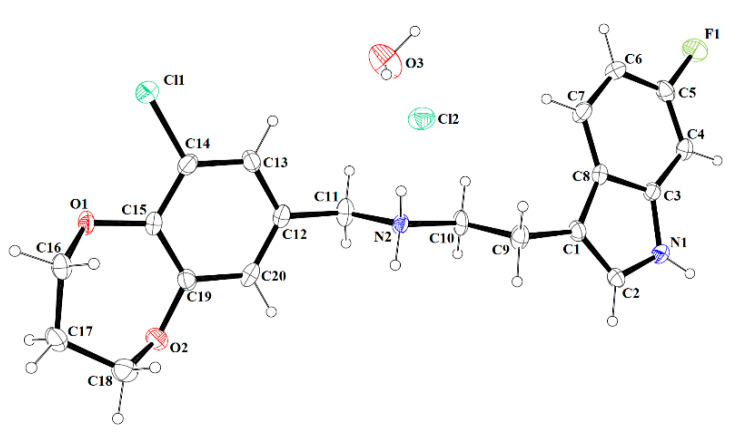
The molecular structure of D2AAK5 with displacement ellipsoids drawn at the 50% probability level.

The single crystal of D2AAK5 was obtained by recrystallization polycrystalline compound from a mixture of ethanol–water (1:2). Crystal data, data collection, and structure refinement details are summarized in Table 1. The molecular structure of the compound is shown in Figure 2, and selected bond distances and angles are listed in Table S1 in Supplementary Information. D2AAK5 is the 1:1 molecular salt with the hydrochloric acid proton transferred to the secondary N atom of N-methylethanamine fragment. The compound crystallizes in centrosymmetric triclinic space group with one molecule in the asymmetric unit. The molecular structure of D2AAK5 compound comprises two fused-ring system (6-fluoro-1*H*-indole and 6-chloro-3,4-dihydro-2*H*-1,5-benzodioxepine units) linked by N-methylethanaminium fragment. As in the previously reported compounds [7,17,18,19], all atoms of the indole group are essentially coplanar (r.m.s. deviation = 0.0115 Å for all non-H atoms), with the largest deviation from the least-squares plane (0.0164(2) Å) being for C8 atom. The dihedral angle between the aromatic and pryrrole ring is 1.5(2)°. The fluorine atom is coplanar with the indole units (r.m.s. = −0.023(3) Å). The 6,7-fused ring system (benzodioxepine) is nonplanar (r.m.s. deviation = 0.2765 Å for all non-H atoms with a maximum deviation of 0.473(2) Å for O(1)). The mean plane of the phenyl ring (C12/C13/C14/C15/C19/C20) is inclined to the plane of dioxepine ring system (C15/O1/C16/C17/C18/O2/C19) by 18.8(2)°. A puckering analysis of the seven-membered ring yielded the parameters q_2_ = 0.271(3) Å, φ_2_ = 204.4(6)°, q_3_ = 0.631(3) Å, and φ_3_ = 129.5(6)°. These parameters indicate that the dioxepine ring adopts a chair conformation [20].

**Figure 3:**
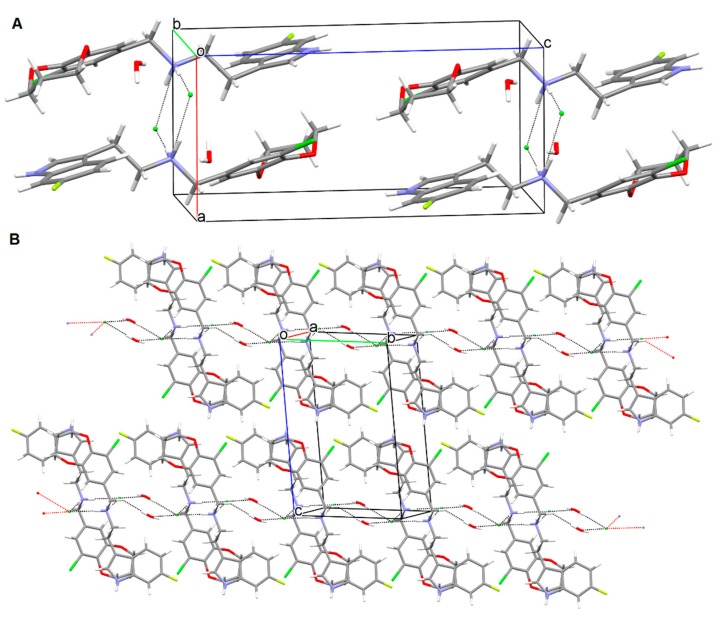
Packing diagrams of D2AAK5: showing inversion dimers with $R_{4}^{2}\left(8\right)$ ring motifs viewed along the *b* axes (**A**); viewed along the *a*-axis showing formation of the ribbons propagating in the *b*-direction (**B**). Hydrogen bonds are indicated by dotted lines.

In the extended structure of D2AAK5, the cations and anions are linked by N2–H2N···Cl2 and N2–H3N···Cl2^ii^ (symmetry code: (ii) −x + 1,−y + 2,−z) hydrogen bonds. These interactions give centrosymmetrical dimers, which form rings with graph-set notation of $R_{4}^{2}\left(8\right)$ [21]. These dimers are shown in Figure 3A. Dimers are joined together through O3–H1W···Cl2 and O3–H2W···Cl2^i^ hydrogen bonds into ribbons propagating in the *b* direction (Figure 3B).

The ribbons are further linked by N1–H1N···O1 hydrogen bonds into sheets propagating in the *bc* plane (Figure S1). The presence of C2-H2···F1 and C11-H11B···O3 interactions stabilize the formed layers whereas the C10-H10B···O3 hydrogen bonds link them together forming three-dimensional structure (Figure S2). The hydrogen bonding and C–H···*Cg* interactions parameters are summarized in Table S2.

### 2.3. Molecular Modeling

**Figure 4:**
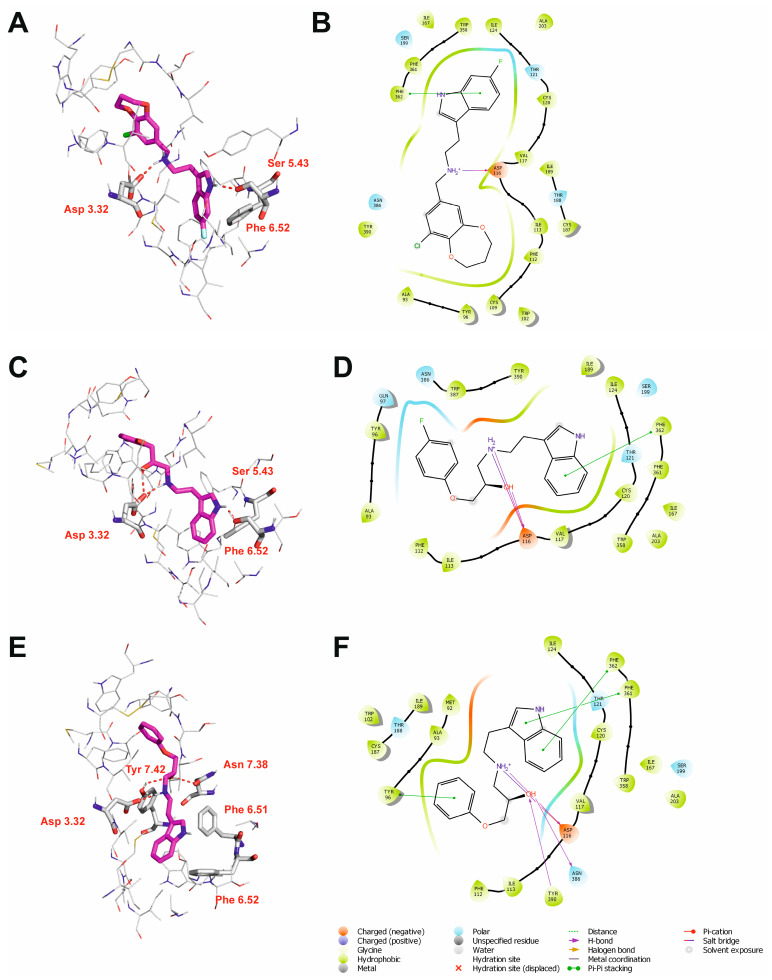
Molecular interactions of D2AAK5 (**A**,**B**), D2AAK6 (**C**,**D**), and D2AAK7 (**E**,**F**) with 5-HT_1A_ receptor based on molecular docking. (**A**,**C**,**E**)—3D details of the binding site. Most important protein residues are shown as sticks with grey carbon atoms. Ligands are shown as sticks with magenta carbon atoms. Non-polar hydrogen atoms were omitted for clarity. Polar interactions are shown as red dashed lines. (**B**,**D**,**F**)—2D overview of the binding site.

**Figure 5:**
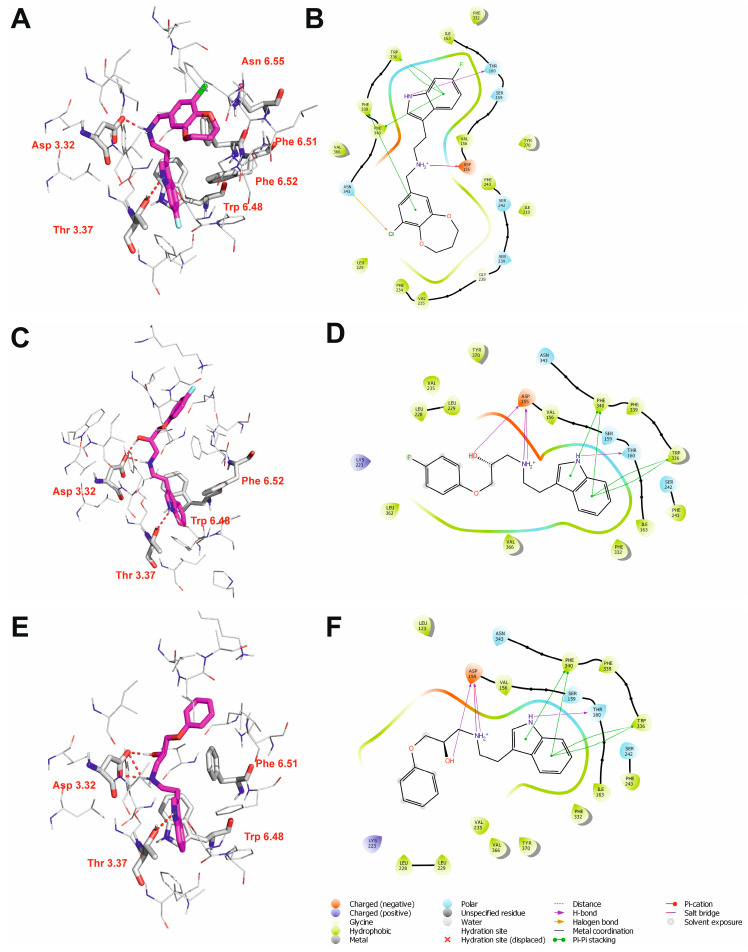
Molecular interactions of D2AAK5 (**A**,**B**), D2AAK6 (**C**,**D**), and D2AAK7 (**E**,**F**) with 5-HT_2A_ receptor based on molecular docking. (**A**,**C**,**E**)—3D details of the binding site. Most important protein residues shown as sticks with grey carbon atoms. Ligands are shown as sticks with magenta carbon atoms. Non-polar hydrogen atoms are omitted for clarity. Polar interactions are shown as red dashed lines. (**B**,**D**,**F**)—2D overview of the binding site.

In order to study interactions of D2AAK5, D2AAK6, and D2AAK7 with serotonin 5-HT_1A_ and 5-HT_2A_ receptors at the molecular level, molecular docking was performed. The selected binding poses for the studied ligands are shown in Figure 4 for 5-HT_1A_ receptor and in Figure 5 for 5-HT_2A_ receptor. All the studied ligands interact with the orthosteric binding pockets of the receptors. The main anchoring point is the conserved Asp 3.32 (Ballesteros-Weinstein numbering), which is involved in electrostatic interaction with the protonatable nitrogen atom of the ligands [22,23]. In case of all ligand-receptor complexes, the indole moiety penetrates deeply into the receptor cavity interacting with the residues of the hydrophobic microdomain typical, i.a., for serotonin and dopamine receptor ligands [24]. These residues include Trp 6.48, Phe 6.51, and Phe 6.52 and hydrophobic interactions with them were observed for many similar ligand–receptor complexes [5,6,8,9,10,25]. Regarding complexes of D2AAK5 and D2AAK6 with serotonin 5-HT_1A_ receptor, an additional hydrogen bond between nitrogen hydrogen from the indole moiety of the ligands and a side chain of Ser 5.43 was observed (see Figure 4A,B or Figure 4C,D, respectively). Concerning interactions of D2AAK7 with serotonin 5-HT_1A_ receptor, additional hydrogen bonds were found between the hydroxyl group of the ligand and the side chains of Asn 7.38 and Tyr 7.42 (see Figure 4 E,F). In case of all ligands interacting with serotonin 5-HT_2A_ receptor additional hydrogen bond is formed between the NH of the indole moiety of the ligands and the side chain of Thr 3.37. Moreover, D2AAK5 was found to form a halogen bond between its chlorine atom and a side chain of Asn 6.55 (see Figure 5A,B).

In order to assess the interaction energy between the studied ligands and receptors, molecular mechanics with generalized Born and surface area solvation (MM/GBSA) calculations were performed (see Table 2). All the ligand–receptor complexes are characterized by the favorable binding energy. The discrepancies between the experimental K_i_ values and the calculated MM/GBSA dG bind energy are in the acceptable range.

Furthermore, 1 μs molecular dynamics (MD) simulations were performed to assess the dynamic aspects of ligand–receptor interactions. First, the root mean square deviation (RMSD) was analyzed (Figure S1 in Supplementary Information). RMSD is used to measure the average change in displacement of a selection of atoms for a particular frame with respect to a reference frame. Ligand RMSD (right Y-axes in Figure S3) indicates how stable the ligand is with respect to the protein and its binding pocket. The observed values of ligand RMSD are significantly smaller than the RMSD of the protein, which indicates that the ligand is relatively stable in its initial binding site (with the exception of ligand D2AAK7 in the serotonin 5-HT_1A_ receptor, see Figure S3C). Moreover, monitoring the RMSD of the protein (left Y-axes in Figure S3) can give insights into its structural conformation throughout the simulation. RMSD analysis can indicate if the simulation has equilibrated—its fluctuations towards the end of the simulation are around some thermal average structure. It can be seen from Figure S3 that protein RMSD is in the range 2–5 Å, which does not indicate a significant conformational change in the proteins, as earlier observed for GPCRs [6,9,10].

Next, the root mean square fluctuation (RMSF) was used for characterization of local changes along the protein chain (Figure S4). The greatest changes were observed for loop regions, as is typical for GPCRs.

**Figure 6:**
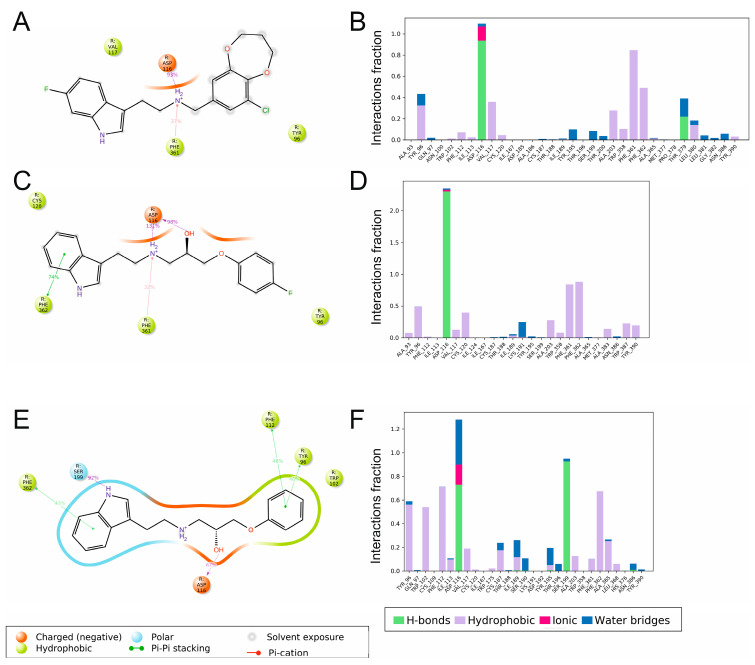
Ligand–receptor contacts (**A**,**C**,**E**) and histograms of ligand–receptor interactions (**B**,**D**,**F**) for serotonin 5-HT_1A_ receptor in complex with D2AAK5 (**A**,**B**), D2AAK6 (**C**,**D**), and D2AAK7 (**E**,**F**) during 1 μs molecular dynamics simulations. The stacked bar charts are normalized over the course of the trajectory: for example, a value of 0.7 suggests that 70% of the simulation time the specific interaction is maintained. Values over 1.0 are possible as some protein residue may make multiple contacts of same subtype with the ligand.

**Figure 7:**
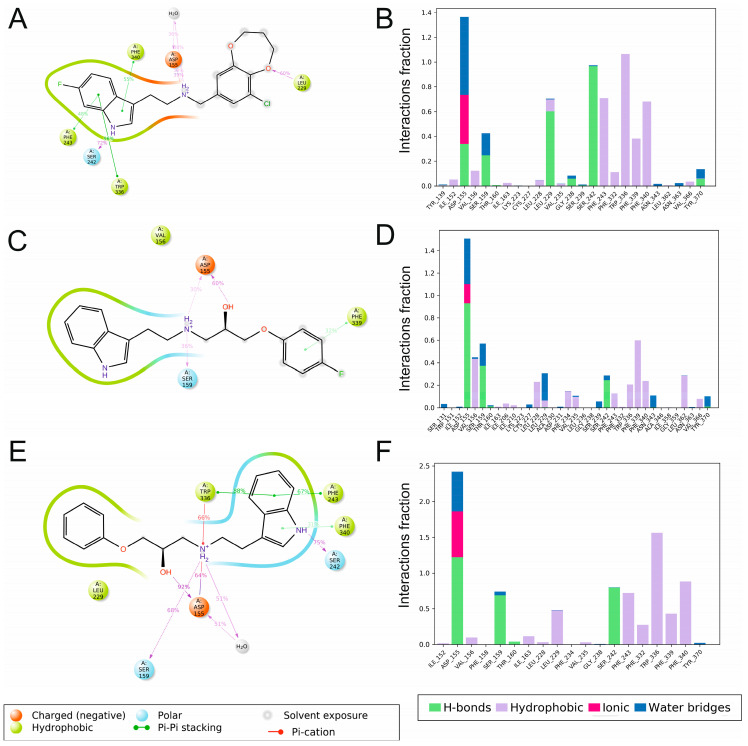
Ligand–receptor contacts (**A**,**C**,**E**) and histograms of ligand–receptor interactions (**B**,**D**,**F**) for serotonin 5-HT_2A_ receptor in complex with D2AAK5 (**A**,**B**), D2AAK6 (**C**,**D**), and D2AAK7 (**E**,**F**) during 1 μs molecular dynamics simulations. The stacked bar charts are normalized over the course of the trajectory: for example, a value of 0.7 suggests that 70% of the simulation time the specific interaction is maintained. Values over 1.0 are possible as some protein residue may make multiple contacts of same subtype with the ligand.

Figure 6 and Figure 7 show ligand–receptor contacts and histograms of ligand–receptor interactions during 1 μs MD simulations for 5-HT_1A_ and 5-HT_2A_ receptor complexes, respectively. In case of D2AAK5 and D2AAK6, the contact between the protonatable nitrogen atom of the ligand and Asp 3.32 is maintained during the entire simulation time (Figure 6A,C, respectively). The contact of D2AAK5 and D2AAK6 with Asp 3.32 is mainly via a hydrogen bond (Figure 6B,D, respectively). In contrast, D2AAK7 diffused from the initial binding site, and the interaction with Asp 3.32 (116) was moved to the hydroxylic group of the ligand (Figure 6E), which is also visible in larger ligand RMSD value (Figure S1C). In case of D2AAK7, the contact with Asp 3.32 is partially mediated via a water bridge (Figure 6F). D2AAK5 and D2AAK6 also maintain a π cation interaction with Phe 6.51 (361) during 37% and 32% of simulations time, respectively. D2AAK6 ligand additionally forms a hydrophobic contact with Phe 6.52 (362) during 74% of simulations. D2AAK7 forms a polar contact with Ser 5.43 (199) during 92% of simulations and hydrophobic contacts with Tyr 2.63 (96), Phe 3.28 (112) and Phe 6.52 (362) during 46%, 46% and 43% of simulations time, respectively.

Regarding complexes of the studied ligands with the serotonin 5-HT_2A_ receptor, the ligand poses are relatively stable (see Figure S3) and the interactions between the protonatable nitrogen atom of the ligand and Asp 3.32 (155) are maintained during 39, 30, and 64% of simulations time, respectively (Figure 7A,C,E). In case of D2AAK6 and D2AAK7 additional interactions with this residue are maintained by the hydroxylic group of the ligand (60% and 92% of simulations time, respectively). In case of all the ligands, the interaction with Asp 3.32 (155) is a mixture of a hydrogen bond, water bridge, and ionic contact. D2AAK5 also forms a polar contact with Ser 5.461 (242, 72% of simulations time), π–π stacking interactions with Phe 5.47 (243, 48% of simulations time), Trp 6.48 (336, 96% of simulations time), and Phe 6.52 (340, 55% of simulations time), as well as a hydrophobic contact with Leu 229 from extracellular loop 2 (ecl2) during 60% of simulations time. In case of D2AAK6 a polar bond with Ser 3.36 (159) is maintained during 36% of simulation times, and π–π stacking interactions with Phe 6.51 (339) are kept during 32% of simulation times.

D2AAK7 forms polar interactions with Ser 3.36 (159) and Ser 5.461 (242) during 68% and 75% of simulations time, respectively. It also maintains a π cation interaction with Trp 6.48 (336, 66% of simulation times) and π–π stacking interactions with Phe 5.47 (243) and Phe 6.52 (340) during 67% and 31% of simulations time, respectively.

### 2.4. Behavioral Studies

#### 2.4.1. Spontaneous Locomotor Activity

**Figure 8:**
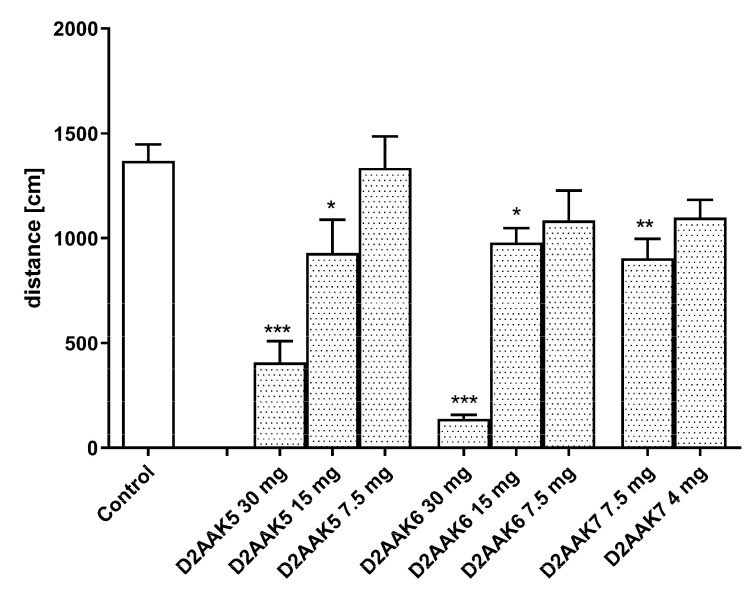
The influence of the tested compounds D2AAK5, D2AAK6 (30 mg, 15 mg and 7.5 mg/kg) and D2AAK7 (7.5 mg and 4 mg/kg) on the spontaneous locomotor activity of mice. Investigated compounds were injected i.p. 60 min before the test. Locomotor activity was measured after 20 min. Data are expressed as mean ± SEM values of the one independent experiment. * *p* < 0.05; ** *p* < 0.01; *** *p* < 0.001 vs. control (Dunnett’s post hoc test).

One-way ANOVA showed significant changes in locomotor activity of mice after administration of the compounds D2AAK5, D2AAK6, and D2AAK7 (F(8, 59) = 16.28; *p* < 0.0001). Dunnett’s post hoc test confirmed a significant decrease in locomotor activity of mice after the administration of compound D2AAK5 and D2AAK6 at the doses of 30 mg/kg (*p* < 0.001) and 15 mg/kg (*p* < 0.05) and D2AAK7 at the dose of 7.5 mg/kg (*p* < 0.01) after 20 min of observation (Figure 8).

#### 2.4.2. Motor Coordination

**Figure 9:**
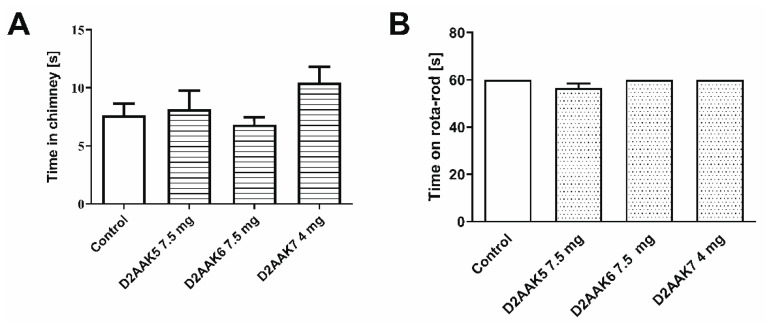
The influence of the tested compounds D2AAK5, D2AAK6 (7.5 mg/kg), and D2AAK7 (4 mg/kg) on motor coordination in mice evaluated in chimney (**A**) and rota–rod (**B**) tests. Investigated compounds were injected i.p. 60 min before the test. Data are expressed as mean ± SEM values of the one independent experiment.

One-way ANOVA did not show any significant changes after administration of the compounds D2AAK5 and D2AAK6 (7.5 mg/kg) and D2AAK7 (4 mg/kg) as evaluated in the chimney (Figure 9A) and in the rota–rod test (Figure 9B).

#### 2.4.3. Effect of Acute Administration of D2AAK5, D2AAK6 (7.5 mg/kg) and D2AAK7 (4 and 2 mg/kg) on Elevated Plus-Maze (EPM) Performance in Mice

**Figure 10:**
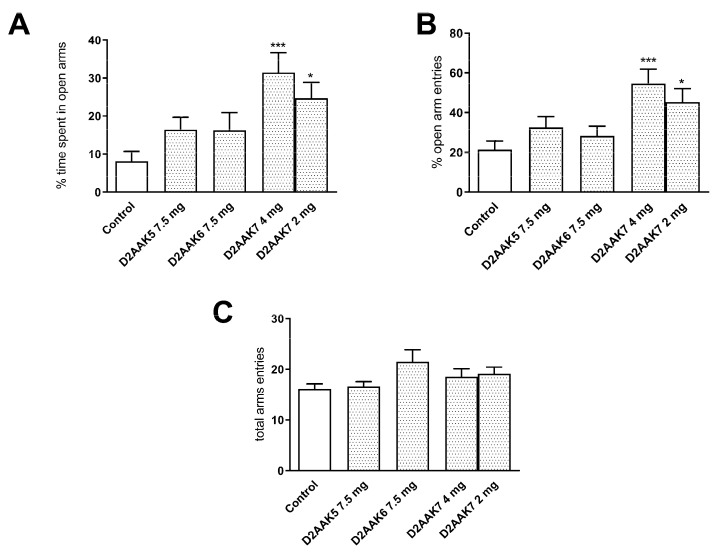
The influence of the investigated compounds D2AAK5, D2AAK6 (7.5 mg/kg), and D2AAK7 (4 and 2 mg/kg) on elevated plus-maze performance in mice–percentage of time spent in open arms (**A**), the percentage of the open arm entries (**B**) and total arm entries (**C**). Investigated compounds were injected i.p. 60 min before the test. The results are expressed as mean ± SEM of the one independent experiment. * *p* < 0.05; *** *p* < 0.001; vs. control (Dunnett’s test).

One-way ANOVA showed significant changes in percentage of time spent in open arms of EPM (F(4, 45) = 4.817; *p* < 0.01 (Figure 10A)) and in the percentage of open arm entries (F(4, 45) = 5.352; *p* < 0.01 (Figure 10B)). There were no significant changes in the total arm entries (F(4, 45) = 1.946; *p*= 0.1192 (Figure 10C)). Dunnett’s post hoc test confirmed a significant increase in time spent in open arms after the administration of compound D2AAK7 at the dose of 4 (*p* < 0.001) and 2 mg/kg (*p* < 0.05). This compound was also able to increase the percentage of open arm entries at the same doses: 4 (*p* < 0.001) and 2 mg/kg (*p* < 0.05). Other compounds remained inactive in this assay.

#### 2.4.4. Effect of Acute Administration of D2AAK5, D2AAK6 (7.5 mg/kg), and D2AAK7 (4 mg/kg) on the Total Duration of Immobility in the Forced Swim Test (FST) in Mice

**Figure 11:**
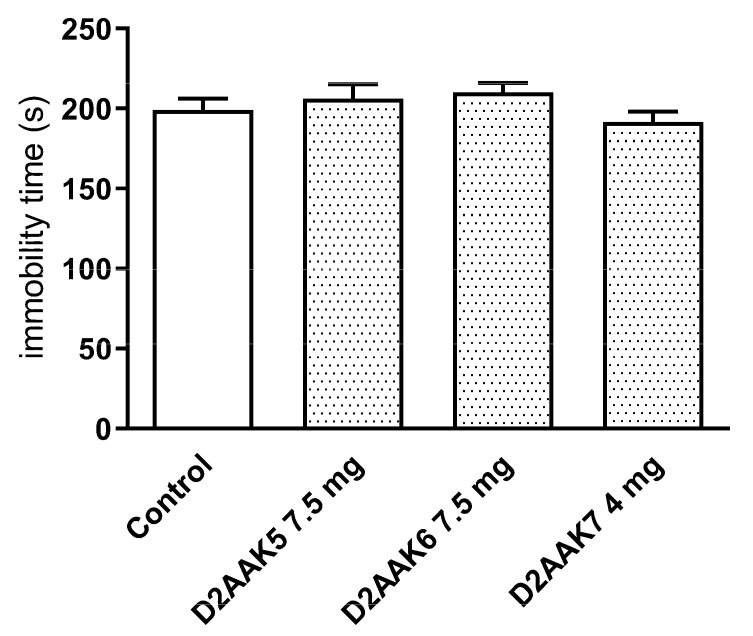
The influence of D2AAK5, D2AAK6 (7.5 mg/kg), and D2AAK7 (4 mg/kg) on the total duration of immobility in the FST in mice. The investigated compounds were administered i.p. 60 min before the test. The values represent means ± SEM of the one independent experiment.

One-way ANOVA showed no significant changes in immobility time after administration of the tested compounds (Figure 11).

#### 2.4.5. Effect of Acute Administration of D2AAK5, D2AAK6 (7.5 mg/kg) and D2AAK7 (4 mg/kg) on Memory Consolidation in Passive Avoidance (PA) Test in Mice

**Figure 12:**
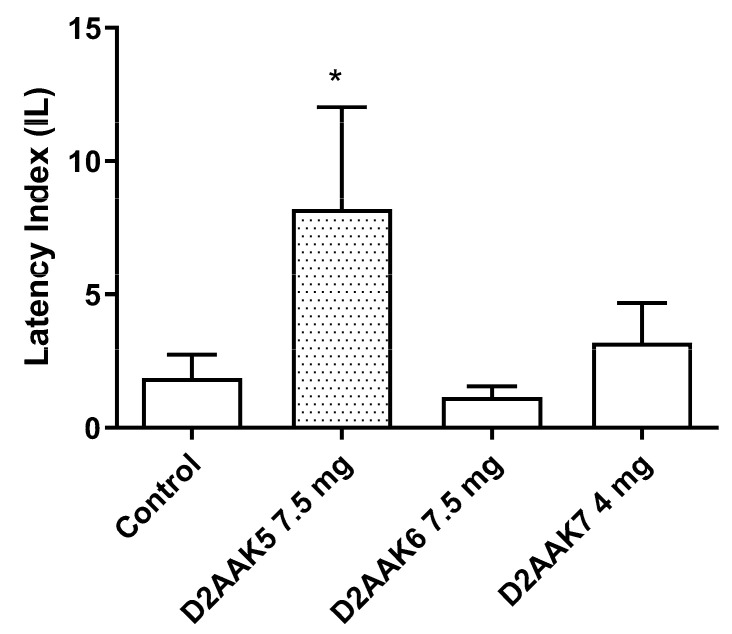
Effect of acute administration of D2AAK5, D2AAK6 (7.5 mg/kg), and D2AAK7 (4 mg/kg) on memory consolidation in the PA test mice. Appropriate groups of mice received injections of D2AAK5, D2AAK6 (7.5 mg/kg), and D2AAK7 (4 mg/kg) and vehicle i.p. on Day 1 immediately after the test and the rodents were retested 24 h later (i.e., on Day 2). The results are expressed as mean ± SEM of the one independent experiment. * *p* < 0.05 vs. control (Student’s *t*-test).

The influence of D2AAK5, D2AAK6 (7.5 mg/kg), and D2AAK7 (4 mg/kg) on memory consolidation was determined during the retention trial of the PA task. Statistical analysis (Student’s *t*-test) confirmed that the acute administration of D2AAK5 at the dose of 7.5 mg/kg, as well as statistically increasing the latency index (IL) values (*p* < 0.05) vs. the vehicle-treated group, indicating its pro-cognitive effect (Figure 12).

## 3. Discussion

The performed investigations were aimed to assess structural and behavioral aspects of ligand–receptor interactions for three ligands previously identified in structure-based virtual screening [5], namely, D2AAK5, D2AAK6, and D2AAK7. These compounds are devoid of dopamine D_2_ receptor affinity, while they are potent ligands of serotonin 5-HT_1A_ and 5-HT_2A_ receptors [5].

The first step of research was to elaborate synthesis of the studied compounds to gain sufficient amount of material for X-ray and behavioral studies. Classical synthetic protocols turned out successful for all the compounds. Next, D2AAK5 was subjected to X-ray studies. Interatomic distances and bond angles of D2AAK5 are in the expected ranges [26] and are comparable to those observed in the related indole [7,17,18,19,27,28] and 3,4-dihydro-2*H*-1,5-benzodioxepine [29,30,31] derivatives.

Next, molecular modeling of the ligand–receptors was performed. Molecular docking revealed the classical binding pose of the ligands in the orthosteric binding pocket of the receptor with Asp 3.32 as the main anchoring point [22,23]. The binding pose of all the ligands in both receptors was similar: the indole moiety of the ligand penetrated deeply into the hydrophobic microdomain of the receptor [24], which was earlier observed for similar ligand–receptor complexes [5,6,8,9,10,25]. MM/GBSA calculations allowed one to evaluate the ligand–receptor binding energy. 1 μs molecular dynamic simulations revealed that the studied compounds are relatively stable in their binding sites with the exception of D2AAK7 in serotonin 5-HT_1A_ receptor.

Compounds D2AAK5, D2AAK6, and D2AAK7 were selected for further functional in vivo studies. The first part of the experiments included: locomotor activity test and motor coordination tests, generally accepted as basic in central activity investigations of new agents [32]. Firstly, compounds D2AAK5 and D2AAK6 were administered at the dose of 30 mg/kg, and they strongly decreased spontaneous motility after 20 min of observation (*p* < 0.001), therefore the dose was reduced twice, and there was also a decrease in the observed values after this dose (*p* < 0.05). The subsequent dilution of both compounds did not significantly affect the mobility, therefore this dose (7.5 mg/kg) was used for other tests. In animals that received D2AAK-7 at a dose of 30 mg/kg, respiratory problems, and loss of righting reflex were observed after a few minutes. After using a half of that dose, some animals still showed the above effects, and after a dose of 7.5 mg/kg, the animals had significantly reduced mobility, hence decreased doses were used in subsequent tests, i.e., 4 and 2 mg/kg, which did not interfere with the locomotor activity of the animals (Figure 8).

Inactive doses in the locomotor test (7.5 mg/kg for D2AAK5 and D2AAK6 and 4 mg/kg for D2AAK7) were used to assess animal coordination in the rota–rod and chimney tests. None of the tested compounds at these doses disturbed the behavior of mice on the rotating rod or extended the time of exit from the chimney compared to the control group (Figure 9), which suggests no effect on muscle tone and no neurotoxic effect.

Anxiety and stress-related disorders are severe mental health conditions that affect the performance of daily tasks and represent a high cost to public health. Charles Darwin’s preliminary observation that animals and humans have similar traits in expressing emotions open the possibility to study the mechanisms of mental disorders in other mammals (mainly rodents). The anxiolytic properties of the new derivatives were investigated in the EPM test. The EPM test is based on the tendency of mice to stay in protected, closed, and dark spaces, as well as their fear of open spaces. Observations describing the reduced percentage of time spent in closed arms of the maze may reflect reduced levels of anxiety in mice. The EPM test is considered to be a precise method of studying changes in anxiety behavior related to the assessment of the therapeutic properties of new drugs [33]. Activity in this test is demonstrated, among others, by buspirone, which belongs to the arylpiperazine derivatives, similarly to the compounds tested at work. It has been confirmed that it is a partial agonist of 5-HT_1A_ serotonin receptors and influences serotonin transmission in the limbic structures of the brain. It has been shown to be effective in generalized anxiety disorder, as well as being less effective in reducing panic [34,35]. It is a drug well tolerated with few side effects. It is also an alternative to benzodiazepines, whose frequent use may lead to the development of tolerance [36]. The results obtained from the experiments carried out in this study showed that only the D2AAK7 compound was active in the EPM test, which statistically significantly increased the time spent in open arms and increased the number of open arm entries. It may be assumed that its anxiolytic effect, as in the case of buspirone, due to its strong affinity, may result from the interaction with the 5-HT_1A_ receptor.

Since all tested compounds are ligands for the 5-HT_1A_ receptor, and D2AAK5 and D2AAK6 have an additional strong affinity for the 5-HT_2A_ receptor, the next step was to test the antidepressant effect of the compounds. There are an increasing number of examples in the literature that blockade of 5-HT_2A_ receptors may increase the therapeutic efficacy of SSRIs (selective serotonin reuptake inhibitors), e.g., risperidone—an atypical 5-HT_2A_ receptor antagonist—reduces symptoms of depression in patients, when treatment is combined with SSRIs and a recognized drug used in clinical practice is trazodone, which also acts as a 5-HT_2A_ receptor antagonist. Studies have reported that it is as effective as other classes of antidepressants, such as SSRIs, and can be used as monotherapy [37].

The antidepressant effect of the new substances was assessed in the FST test, which is a simple and fast test established in experimental pharmacology for detecting the antidepressant effect of tested substances [38]. The animals were placed in the water, and the depressive behavior was observed as immobility and floating of the rodent in the water with only slight movements. In this test, drugs with an antidepressant effect shorten the time of immobility, and those with depressive properties prolong it [39]. It is designed to reflect human depression. SSRIs are an example of antidepressants active in this test. They block the serotonin transporter (SERT) protein, which transports serotonin, and reduce its reuptake from the synaptic cleft. Due to their effectiveness, they are one of the most commonly used drugs in depression. After administration of D2AAK5, D2AAK6, and D2AAK7, no antidepressant activity was shown in mice because the time of immobility was almost the same as in the control group.

Mental illnesses, such as depression, are often associated with impaired cognitive functions, learning, and memory. Additionally, in this context, arylpiperazine derivatives seem to be good candidates. It has been shown, inter alia, that the well known antidepressant vortioxetine, also characterized by the structure of arylpiperazine, has an antidepressant and anxiolytic effect, and is also pro-cognitive [40]. In this study, it was therefore decided to test the pro-cognitive properties of new substances in the PA test. It is known that rodents intuitively prefer being in darker places. In the study, animals were subjected to electric pulses in a chamber with no access to light. This was to teach them that being in this place is a threat. In further observations, the mice treated in this way should spend more time in a bright room, as this zone will be associated with safety. The D2AAK5 compound caused an increase in the latency index—the time of transition to the darker chamber was longer than in the case of the control, which may suggest that it stimulates memory processes. Confirmation of the observed activity also in other memory tests could offer hope for the treatment of mental illnesses associated with dementia. It can be speculated that the mechanism of action of the D2AAK5 compound is related to the influence on the 5-HT_1A_ receptor, which is known to be involved in the modulation of memory in the brain [41].

The demonstrated anxiolytic effect for D2AAK7 (probably related to the interaction with 5-HT_1A_ receptors) and the improvement of memory processes in the case of D2AAK5 (probably related to the interaction with 5-HT_1A_ and 5-HT_2A_ receptors) suggest further testing of these activities in advanced animal models to confirm and better understand the underlying mechanism.

## 4. Materials and Methods

### 4.1. Chemistry

#### 4.1.1. General

NMR spectra were recorded on a Bruker AVANCE III 600 MHz instrument equipped with a BBO Z-gradient probe using DMSO-*d_6_* as solvent. All chemical shifts are reported in parts per million (δ), from tetramethylsilane (0 ppm), and are referenced to the residual proton in the respective solvent: DMSO-*d_6_* (2.49 ppm) for ^1^H NMR and DMSO-*d_6_* (39.5 ppm) for ^13^C NMR. Coupling constants (J) are reported in Hertz (Hz). HRMS analyses were performed on a Bruker microTOF-Q II mass spectrometer in electrospray mode with a time-of-flight (TOF) mass analyzer using acetonitrile as solvent. IR spectra were recorded on Thermo Nicolet 6700 instrument equipped with ATR. Obtained data were processed using MestReNova v.14.0.0 (see Suplementary Information). ^1^H and ^13^C NMR spectra, as well as HRMS and IR spectra of the final compounds, are shown in Supplementary Information.

#### 4.1.2. Synthesis of 5-Chlorovanillin

Vanillin (7.61 g, 50 mmol) and NCS (6.68 g, 50 mmol) were dissolved in glacial acetic acid (100 mL). The reaction was stirred for 12h at room temperature. Formed precipitate was filtered, washed with glacial acetic acid, and allowed to dry. The product was obtained as white solid (4.45 g, 48%).

#### 4.1.3. Synthesis of 3-Chloro-4,5-dihydroxybenzaldehyde

5-chlorovanillin (1.87 g, 10 mmol) and aluminium chloride (1.47 g, 11 mmol) were dissolved in dichloromethane (100 mL). Pyridine (3.24 mL, 40 mmol) was added dropwise, and the reaction was stirred for 12h at room temperature. Hydrochloric acid (20%, 50 mL) was added, and the mixture was diluted with water (50 mL). Subsequently, it was extracted three times with ethyl acetate. The organic phase was evaporated to yield a product as a grey solid (1.63 g, 94%).

^1^H NMR (600 MHz, DMSO-*d6*) δ 10.43 (s, 2H), 9.70 (s, 1H), 7.43 (d, *J* = 1.9 Hz, 1H), 7.22 (d, *J* = 1.9 Hz, 1H). ^13^C NMR (151 MHz, DMSO-*d6*) δ 191.1, 148.7, 147.3, 128.8, 124.7, 120.7, 112.9.

#### 4.1.4. Synthesis of 9-Chloro-3,4-dihydro-2H-benzo[b][1,4]dioxepine-7-carbaldehyde **4**

3-Chloro-4,5-dihydroxybenzaldehyde (1.63 g, 9.5 mmol), 1,3-dibromopropane (1.90 mL, 10.4 mmol), and caesium carbonate (7.69 g, 24 mmol) were dissolved in DMF (40 mL). The reaction mixture was stirred at 60° C for 3h, and it was diluted with water afterwards. The solution was extracted three times with ethyl acetate. The layers were separated, and the organic phase was concentrated on a rotavap. The oily residue was purified by dry column vacuum chromatography using 20% ethyl acetate in hexane as an eluent, to yield the product as a white solid (990 mg, 49%).

^1^H NMR (600 MHz, CDCl_3_) δ 9.82 (s, 1H), 7.60 (d, *J* = 2.0 Hz, 1H), 7.40 (d, *J* = 2.0 Hz, 1H), 4.49 (t, *J* = 6.0 Hz, 2H), 4.35 (t, *J* = 6.0 Hz, 2H), 2.33 (*p*, *J* = 5.9 Hz, 2H). ^13^C NMR (151 MHz, CDCl_3_) δ 189.6, 152.5, 152.0, 131.2, 126.8, 125.7, 121.3, 70.8, 70.4, 30.3.

#### 4.1.5. Synthesis of 6-Fluoro-1H-indole-3-carbaldehyde

POCl_3_ (1.72 mL) was mixed with DMF (8.60 mL) by dropwise additional with cooling in the ice bath. 6-fluoroindole (1.00 g 7.4 mmol) was dissolved in DMF (10 mL) and added to the previously prepared DMF-phosphoryl trichloride mixture. The mixture was stirred for 3 h, and the 4M potassium hydroxide (18.50 mL) was added. It was stirred at reflux for 2 h. Upon completion (TLC analysis), the reaction mixture was cooled, and a saturated solution of sodium bicarbonate (20 mL) was added. The solution was extracted three times with ethyl acetate. The separated organic fraction was concentrated under reduced pressure to yield the product as a white solid (1 g, 83%).

#### 4.1.6. Synthesis of 6-Fluoro-3-(2-nitrovinyl)-1H-indole

6-Fluoro-1*H*-indole-3-carbaldehyde (0.82 g, 5 mmol) was added to isopropanol (5 mL) and stirred at 60 °C until full dissolution. Nitromethane (540 µL, 10 mmol), acetic acid (143 µL, 2.5 mmol), and butylamine (50 µL, 500 µmol) were added to the mixture in succession. The reaction mixture was heated at 60 °C for 3h. After cooling, the orange crude was precipitated from the reaction mixture. The crude was filtered, washed with isopropanol, and dried (660 mg, 66%).

^1^H NMR (600 MHz, DMSO-*d6*) δ 12.26 (s, 1H), 8.39 (d, *J* = 13.4 Hz, 1H), 8.26 (s, 1H), 8.04 (d, *J* = 13.4 Hz, 1H), 8.02 (dd, *J* = 8.8, 5.2 Hz, 1H), 7.34 (dd, *J* = 9.5, 2.4 Hz, 1H), 7.09 (ddd, *J* = 9.7, 8.7, 2.4 Hz, 1H). ^13^C NMR (151 MHz, DMSO-*d6*) δ 160.8, 159.2, 138.4, 138.3, 137.4, 134.7, 132.1, 122.3, 122.3, 121.8, 110.6, 110.4, 108.7, 99.7, 99.5.

#### 4.1.7. Synthesis of 6-Fluorotryptamine **5**

1 M LiAlH_4_ (12.00 mL, 11.70 mmol) was diluted with anhydrous THF (40 mL), and 6-Fluoro-3-(2-nitrovinyl)-1*H*-indole (0.6 g, 3 mmol) was added. The reaction mixture was stirred at reflux for 12 h. After cooling, concentrated Rochelle salt solution (50 mL) was added to the reaction mixture. The solution was extracted three times with ethyl acetate. The phases were separated, and the organic phase was evaporated. The brown oily residue was purified by dry column vacuum chromatography, using 5% 7M NH_3_ in MeOH/DCM as an eluent. The product was obtained as a solid (200 mg, 42%).

^1^H NMR (600 MHz, CDCl_3_) δ 8.24 (s, 1H), 7.50 (dd, *J* = 8.7, 5.3 Hz, 1H), 7.03 (dd, *J* = 9.7, 2.3 Hz, 1H), 7.01–7.00 (m, 1H), 6.88 (ddd, *J* = 9.6, 8.7, 2.3 Hz, 1H), 3.02 (t, *J* = 6.7 Hz, 2H), 2.88 (t, *J* = 6.6 Hz, 2H), 1.53 (s, 2H). ^13^C NMR (151 MHz, CDCl_3_) δ 160.8, 159.3, 136.4, 136.3, 124.1, 122.2, 122.2, 119.6, 119.5, 113.9, 108.1, 108.0, 97.5, 97.3, 42.3, 29.4.

#### 4.1.8. Synthesis of N-((9-Chloro-3,4-dihydro-2H-benzo[b][1,4]dioxepin-7-yl)methyl)-2-(6-fluoro-1H-indol-3-yl)ethan-1-amine 1 (D2AAK5)

The reaction was carried out under anhydrous conditions. 6-fluorotryptamine (0.356 g, 2 mmol) was dissolved in MeOH (20 mL) with addition of Na_2_SO_4_ (1.00 g). Aldehyde **4** (0.53 g, 2.5 mmol) was added and the mixture was stirred for 4 h. Subsequently sodium borohydride (0.12 g, 3 mmol) was added, and the reaction was stirred for 12h at room temperature. The reaction mixture was quenched with water (50 mL) and extracted three times with DCM. The phases were separated, and the organic phase was concentrated under reduced pressure. The oily product was converted to hydrochloride salt, which was crystallized from ethanol–ether mixture. Pure product was filtered, washed with ethanol-ether mixture, and dried to yield white solid (377 mg, 44%). M.p. (HCl salt) 197–199 °C with decomposition. HRMS (ESI) calc. (M + H)^+^ 375.1270 exp. (M + H)^+^ 375.1286

^1^H NMR (600 MHz, DMSO-*d6*) δ 11.09 (s, 1H), 9.46 (s, 2H), 7.58 (dd, *J* = 8.7, 5.3 Hz, 1H), 7.40 (s, 1H), 7.23 (dd, *J* = 7.3, 2.3 Hz, 2H), 7.15 (dd, *J* = 10.1, 2.4 Hz, 1H), 6.87 (td, *J* = 9.2, 2.3 Hz, 1H), 4.23 (t, *J* = 5.5 Hz, 2H), 4.19 (t, *J* = 5.4 Hz, 2H), 4.08 (s, 2H), 3.11 (s, 4H), 2.16 (*p*, *J* = 5.5 Hz, 2H). ^13^C NMR (151 MHz, DMSO-*d6*) δ 160.2, 158.6, 152.4, 148.1, 136.6, 136.5, 127.9, 126.1, 125.6, 124.4, 124.1, 122.9, 119.7, 119.6, 110.1, 107.5, 107.3, 98.1, 97.9, 71.4, 71.1, 49.0, 47.2, 31.3, 22.0.

#### 4.1.9. Synthesis of 1-((2-(1H-Indol-3-yl)ethyl)amino)-3-(4-fluorophenoxy)propan-2-ol 2 (D2AAK6)

Tryptamine (0.80 g, 5 mmol) and [(4-fluorophenoxy)methyl]oxirane (0.84 g, 5 mmol) were dissolved in isopropanol (20 mL). Reaction mixture was stirred at reflux overnight. After evaporation oily residue was purified by DCVC using a 5% NH_3_-MeOH in DCM as an eluent, to yield yellow-green oil. The combined fractions were evaporated, yielding an oily product. The crude was converted into hydrochloride salt, which was purified by crystallization from ethanol. After filtration, pure product was washed with ethanol and dried, yielding white solid (328 mg, 20%). M.p. (HCL salt) 189–191 °C with decomposition. HRMS (ESI) calc. (M + H)^+^ 329.1660 exp. (M + H)^+^ 329.1673

^1^H NMR (600 MHz, CD_3_OD) δ 7.62 (dt, *J* = 7.9, 1.0 Hz, 1H), 7.38 (dt, *J* = 8.2, 0.9 Hz, 1H), 7.21 (s, 1H), 7.13 (ddd, *J* = 8.1, 7.1, 1.1 Hz, 1H), 7.05 (ddd, *J* = 8.0, 7.0, 1.0 Hz, 1H), 7.02–6.97 (m, 2H), 6.92–6.87 (m, 2H), 4.27 (dtd, *J* = 8.3, 5.1, 3.2 Hz, 1H), 4.01–3.93 (m, 2H), 3.40–3.35 (m, 2H), 3.33–3.30 (m, 1H), 3.25–3.16 (m, 3H). ^13^C NMR (151 MHz, CD_3_OD) δ 159.7, 158.1, 156.1, 156.1, 138.3, 128.2, 124.3, 122.8, 120.1, 119.0, 116.9, 116.9, 116.8, 116.7, 112.6, 110.2, 71.8, 66.6, 51.3, 49.6, 23.2.

#### 4.1.10. Synthesis of 1-((2-(1H-Indol-3-yl)ethyl)amino)-3-phenoxypropan-2-ol 3 (D2AAK7)

To the solution of 2-(phenoxymethyl)oxirane (125 mg, 832 μmol) in isopropanol (10 mL), tryptamine was added (133.5 mg, 833 µmol), and the reaction mixture was stirred at room temperature for 1h. After evaporation, the oily product was converted into the hydrochloride salt, which was purified by crystallization from ethanol. After filtration, the pure product was washed with ethanol–ether mixture and dried to yield a white solid (498 mg, 28%). M.p. (HCl salt) 196–197 °C with decomposition. HRMS (ESI) calc. (M + H)^+^ 311.1754 exp. (M + H)^+^ 311.1763

^1^H NMR (300 MHz, DMSO-*d6*) δ 11.04 (s, 1H), 9.37 (s, 1H), 9.06 (s, 1H), 7.62 (d, *J* = 7.7 Hz, 1H), 7.43–7.19 (m, 4H), 7.16–6.88 (m, 5H), 5.96 (d, *J* = 4.9 Hz, 1H), 4.35–4.22 (m, 1H), 4.06–3.91 (m, 2H), 3.32–2.97 (m, 6H). ^13^C NMR (75 MHz, DMSO-*d6*) δ 158.73, 136.72, 129.99, 127.25, 123.64, 121.63, 121.28, 118.90, 118.72, 114.97, 112.01, 109.91, 70.15, 65.41, 50.02, 48.14, 22.03.

### 4.2. X-ray Studies

Data for D2AAK5 (MoK*_α_*) were collected on an Oxford Diffraction Xcalibur CCD diffractometer using the program CrysAlis [42]. Programs (implemented in WinGX software [43]) were used for structure solution (direct methods, SHELXS-2018) and refinement (SHELXL-2018) [44]. Non-hydrogen atoms were refined with the anisotropic displacement parameters. Hydrogen atoms attached to carbon were placed in calculated positions and refined using a riding model with C–H = 0.95 Å (aromatic) and 0.99 Å (methylene group) with U_iso_(H) = 1.2 Ueq(C). The N−H hydrogen atoms were located on a difference-Fourier map and freely refined. Hydrogen atoms at the water molecule were also located on a difference Fourier map. Positions of these H atoms were not refined (AFIX 3), and their isotropic temperature parameters were set to be 1.5 times greater than U_iso_ of the corresponding O atom. The ORTEP3 [43] and Mercury [45] programs were used to prepare the molecular graphics. The PLATON program were used for geometrical calculations [46]. The CIF files have been deposited in the Cambridge Crystallographic Data Center (CCDC). These data can be obtained free of charge from The Cambridge Crystallographic Data Centre via www.ccdc.cam.ac.uk/data_request/cif (accessed on 26 December 2022) (or from the CCDC, 12 Union Road, Cambridge CB2 1EZ, UK; Fax: +44-1223-336033; E-mail: deposit@ccdc.cam.ac.uk).

### 4.3. Molecular Modeling

#### 4.3.1. Ligand Preparation

The 3D structures of D2AAK5, D2AAK6 and D2AAK7 were modelled using LigPrep module [47] of Schrödinger suite of software, v. 2019-4 as previously reported [7,8]. For D2AAK6 and D2AAK7 ligands, which possess a chiral carbon atom, R enantiomer was arbitrarily taken for calculations. In case of D2AAK5, its X-ray structure was used as a starting conformation. In order to sample ligand protonation state Epik module [48] of Schrödinger suite of software, v. 2019-4 was applied.

#### 4.3.2. Protein Preparation

X-ray structure of serotonin 5-HT_2A_ receptor in inactive conformation in complex with an antagonist risperidone (PDB ID: 6A93 [49]) and cryo-EM structure serotonin 5-HT_1A_ receptor in active conformation in complex with a partial agonist aripiprazole (PDB ID: 7E2Z [50]) were used as previously reported [7,8]. The structures of the receptors were preprocessed using the Protein Preparation Wizard of Maestro Release 2019.4 [51], as previously reported [7,8]. Yasara Structure v. 20.12.24 [52] tool for loop modelling was used to build receptors extracellular loops if necessary.

#### 4.3.3. Molecular Docking and MM/GBSA Calculations

Standard precision (SP) molecular docking technique with Glide [53] from Schrödinger release 2019-4 was applied. The grid files were obtained based on co-crystallized ligands in respective experimental structures. Most important hydroxyl groups of the protein residues in the binding pocket were made flexible, as previously reported [7,8]. An amount of 100 poses were obtained for each ligand–receptor complex. The poses were then filtered to keep only those where interaction of the conserved Asp3.32 (Ballesteros-Weinstein numbering [54]) of the receptor with a protonatable nitrogen atom of the ligand was maintained. The final poses were selected based on Glide docking scores and the visual inspection. Maestro Release 2019.4 [51] and PyMol 2.0.4 [55] were applied for molecular modeling results visualization. Prime MM/GBSA [56] of Schrödinger suite of software v. 2019-4 was used to estimate the relative binding affinity of protein–ligand without taking into account of any simulation process. The flexible residue distance was defined for 5 Å.

#### 4.3.4. Molecular Dynamics Simulations

Molecular dynamics with Desmond v. 3.0.3.1 [57] were performed for the ligand–receptor complexes, as described previously [9,10]. Shortly, the systems were embedded in POPC (1-palmitoyl-2-oleoyl-sn-glycero-3-phosphocholine) membrane, hydrated, and ions were added for protein charges neutralization and then added to a concentration of 0.15 M NaCl. The TIP3P model for waters was used. The complexes were minimized and subjected to MD. The production runs (1 μs) were performed in NPT ensemble with no restrictions.

### 4.4. Behavioral Studies

#### 4.4.1. General Procedures

All experiments were conducted on 6-week-old naive male Swiss mice weighing 24–30 g in the Experimental Medicine Center, Medical University of Lublin, Poland. The animals were housed (five per cage) under standard laboratory conditions (room temperature of 22 ± 1 °C and a relative humidity of 50–60% during a 12/12 h light-dark cycle, lights on at 8:00). They had free access to laboratory food-pellet diet LSM, Agropol Motycz, Poland and tap water, except for the short time that they were removed from their cages for testing. All experiments were performed in strict accordance with the EU Directive 2010/63/EU for animal experiments and with the approval of the Local Ethics Committee for Animal Experimentation in Lublin (license number 55/2022). Mice were randomized for groups and treatments. The investigated compounds (D2AAK5, D2AAK6 and D2AAK7) in all tests were administered intraperitoneally (i.p.), dissolved in dimethyl sulfoxide (DMSO, final concentration of 0.1%) and then diluted by aqueous solution of 0.5% methylcellulose (tylose) and injected 60 min before the tests in a volume of 10 mL/kg. Control groups received tylose injections of the same volume and via the same route of administration.

#### 4.4.2. Spontaneous Locomotor Activity

The automatic device-actimeters Opto-Varimex-4 Auto-Track (Columbus Instruments, OH, USA) was used to measure the locomotor activity of experimental animals. The mice were individually placed in the cages for 30 min: first for 10 min of acclimatization and a further 20 min of observation and the distance (in centimeters) traveled during this time was measured. After each mouse, the cages were cleaned with 10% ethanol.

#### 4.4.3. Motor Coordination

The effects of D2AAK5, D2AAK6, and D2AAK7 compounds were also measured in the rota–rod [58] and chimney [59] tests. In the first test, the ability of mice to balance on a rotating rod (18 rpm, constantly) was assessed for 1 min. The second test is carried out using a polymer tube (named chimney) with an internal diameter of 3 cm and a length of 25 cm. The chimney is placed in a horizontal position, and the animal is led inside. Once the mouse is at the other end, the tube is placed vertically, forcing the rodent to escape only by climbing backwards on the rough surface inside the tube. Correct motor coordination is considered to be lowering the chimney in no more than 60 s. Prior to the study, animals were trained for 3 days, and only those that were able to stay on the rotating rod or leave the chimney within 60 s were included.

#### 4.4.4. EPM Test

Anxiety behaviors were measured using the EPM test according to the Lister method [60]. The device was made up of four black-painted, crossed arms arranged in a plus with a central platform (5 cm × 5 cm). Two arms 30 × 5 cm were open, while the other two, 30 cm × 5 cm × 15 cm, were closed. Individual types of arms were placed opposite each other. The whole structure was elevated to a height of 38.5 cm above the floor and lit with weak red, matte light. The experiment was conducted in a quiet, dark room: the mice were individually placed at the central square of the maze, facing the open arm, and their behavior was observed for 5 min by an observer equipped with a stopwatch, unaware of the treatment schedule. The time spent in the EPM open arms, the number of entries into the open arms, and the total number of entries into both types of arms were measured.

#### 4.4.5. FST in Mice

The study was carried out using the test proposed by R. Porsolt [61]. The method is based on the observation of an animal forced to swim in a confined space from which there is no escape. After an initial period of vigorous attempts to escape, the animal finally gives up on escaping. The test consists of immersing the mouse individually in a cylindrical beaker (diameter 10 cm, height 25 cm) filled with water (at a temperature of 23–25 °C) to a height of 10 cm for 6 min. The immobility time, considered as the state in which the mouse performs only those movements necessary to keep its head above the water, is measured between 2 and 6 min.

#### 4.4.6. PA Task

Memory-related effects were measured by the PA test, commonly used to examine different stages of memory [62]. The PA apparatus is divided into two compartments connected by a guillotine door. One chamber of the apparatus is illuminated with bright fluorescent light (8 W) and connected with the second-dark chamber, equipped with an electric grid floor. The procedure has been previously described [7]. Briefly: on training day (pre-test), mice were placed individually into the bright chamber and allowed to freely explore it for 30 s. After this time, the guillotine door was lifted to allow the mice to enter the dark chamber, which in turn was punished with an electric foot shock (0.15 mA for 2 s); this phase lasted a maximum of 5 min. On that day, the latency time for entering to the dark compartment (TL1) was measured and, immediately after that, the animals were administered with the tested compounds D2AAK5, D2AAK6 (7.5 mg/kg; i.p.), D2AAK7 (4 mg/kg), or vehicle (control group). To measure memory consolidation, on test day (24 h later), the same mice were retested in the PA apparatus, except for the delivery of an electric foot shock, and TL2 was recorded. If the animal did not enter the dark compartment during this test, TL2 was estimated as 300 s [53,54].

The changes in PA performance were expressed as the difference between pre-test and test latencies and was taken as a latency index (LI). Changes in PA performance were expressed as the difference between pre-test and test latencies and designated as latency index (LI) expressed by the formula:

$$LI=\frac{TL1-TL2}{TL1}$$

where TL1-time needed to move to the dark camber during a training day, TL2-time needed to re-enter dark compartment, measured 24 h later.

#### 4.4.7. Statistical Analysis

The results were calculated by the one-way analysis of variance ANOVA, followed by Dunnett’s post-hoc test and Student’s *t*-test, if needed. The results are presented as mean  ±  standard errors (SEM). The level of *p*  < 0.05 was considered as statistically significant. All the figures were prepared by the GraphPad Prism version 5.00 for Windows, GraphPad Software (San Diego, CA, USA; www.graphpad.com).

## 5. Conclusions

Here we present synthesis and detailed structural and behavioral studies of compounds D2AAK5, D2AAK6, and D2AAK7 previously identified in structure-based virtual screening. X-ray studies of D2AAK5 compound allowed one to determine its stable conformation in the solid state and supplied the starting conformation for molecular modeling. Molecular docking revealed an interaction mode typical for orthosteric ligands of aminergic GPCRs with the conserved Asp 3.32 as the main anchoring point. Moreover, it was found that all the ligands adopt a similar binding pose in the studied receptor with the indole moiety penetrating deep into the hydrophobic microdomain of the proteins. The obtained ligand receptor complexes were subjected to MM/GBSA calculations to evaluate the binding energy and to 1 μs MD simulations to evaluate their stability. All the ligand–receptor complexes, with the exception of D2AAK7 in the 5-HT_1A_ receptor, were relatively stable in MD simulations. In behavioral studies in mice models, D2AAK7 exerts anxiolytic activity in the EPM test, while D2AAK5 has a beneficial effect on the memory processes in the PA test. In summary, the performed studies provide information for the design of more potent analogues of the studied compounds.

## Figures and Tables

**Table 1:** Crystal data and structure refinement for D2AAK5.

Formula	C_20_H_23_Cl_2_FN_2_O_3_
Formula weight	429.30
Temperature K	120(2)
Crystal system	triclinic
Space group	*P* $\overset{-}{1}$
a (Å)	8.0886(9)
b (Å)	9.0019(7)
c (Å)	14.9140(15)
α (°)	82.422(7)
β (°)	83.707(9)
γ (°)	65.926(9)
Volume (Å^3^)	980.9(2)
Z	2
Calculated density (g cm^−3^)	1.453
μ (mm^−1^)	0.365
Absorption correction	multi-scan
F(000)	448
Crystal size (mm)	0.40 × 0.30 × 0.20
θ range (°)	2.490 to 26.727
Limiting indices	−8 ≤ h ≤ 10, −7 ≤ k ≤ 11, −18 ≤ l ≤ 18
Reflections collected/unique	7548/4168
R_int_	0.0436
Data/restraints/parameters	4168/0/261
GooF (F^2)^	1.041
Final R indices [I > 2σ(I)]	R_1_ = 0.0547, wR_2_ = 0.1055
R indices (all data)	R_1_ = 0.0927, wR_2_ = 0.1241
Largest diff. peak/hole, e Å^−3^	0.356/−0.289
CCDC No.	2223764

**Table 2:** Affinity of the studied ligands and MM/GBSA binding energy to serotonin 5-HT_1A_ and 5-HT_2A_ receptors.

	5-HT_1A_ Receptor		5-HT_2A_ Receptor	
Ligand	K_i_ [nM] [5]	MM/GBSA dG Bind kcal/mol	Ki [nM] [5]	MM/GBSA dG Bind kcal/mol
D2AAK5	938 ± 41	−40.38	135 ± 81	−52.27
D2AAK6	115 ± 29	−61.03	246 ± 99	−67.91
D2AAK7	88.5 ± 14.4	−48.89	546 ± 194	−65.83

## Data Availability

Not applicable.

## Supplementary Materials

The following supporting information can be downloaded at: https://www.mdpi.com/article/10.3390/molecules28010383/s1, Figure S1: Packing diagrams of D2AAK5 showing formation sheets viewed along the *a*-axis. Hydrogen bonds are indicated by dotted lines; Figure S2: The 3D supramolecular structure of the D2AAK5 constructed by the connection of adjacent sheets through C10–H1B∙∙∙O2 hydrogen bonds. The hydrogen bonds shown as dotted lines. Hanging contact are omitted for clarity; Table S1: Interatomic distances and selected bond angles for D2AAK5; Table S2: Hydrogen bonding and C-H∙∙∙Cg interactions geometry; Figure S3: Ligand (purple) and protein C_α_ RMSD during 1 μs MD simulations for D2AAK5-5HT_1A_ (A), D2AAK6-5HT_1A_ (B), D2AAK7-5HT_1A_ (C), D2AAK5-5-HT_2A_ (D), D2AAK6-5HT_2A_ (E) and D2AAK7-5HT_2A_ (F) ligand-receptor systems; Figure S4: Protein Cα RMSD during 1μs MD simulations for D2AAK5-5HT_1A_ (A), D2AAK6-5HT_1A_ (B), D2AAK7-5HT_1A_ (C), D2AAK5-5-HT_2A_ (D), D2AAK6-5HT_2A_ (E) and D2AAK7-5HT_2A_ (F) ligand-receptor systems; ^1^H and ^13^C NMR spectra of the investigated compounds.

## Sample Availability

Samples of the compounds are available from the authors.
