# Supplementary material for: Synthesis, Structural and Behavioral Studies of Indole Derivatives D2AAK5, D2AAK6 and D2AAK7 as Serotonin 5-HT1A and 5-HT2A Receptor Ligands
Source: Molecules. 2023 Jan 2;28(1):383. doi: 10.3390/molecules28010383 (PMC9823611; doi:10.3390/molecules28010383)

Supplementary Information

Synthesis, structural and behavioral studies of D2AAK5, D2AAK6 and D2AAK7 as serotonin 5-HT1A and 5-HT2A receptor ligands

Agnieszka A. Kaczor [1,2*], Ewa Kędzierska [3], Tomasz M. Wróbel [1], Angelika Grudzińska [1], Angelika Pawlak [1], Tuomo Laitinen[2] and Agata Bartyzel [3]

[1] Department of Synthesis and Chemical Technology of Pharmaceutical Substances with Computer Modeling Laboratory, Faculty of Pharmacy, Medical University of Lublin, 4A Chodźki St., PL-20093 Lublin, Poland
[2] School of Pharmacy, University of Eastern Finland, FI-70211 Kuopio, Finland
[3] Department of Pharmacology and Pharmacodynamics, Faculty of Pharmacy, Medical University of Lublin, 4A Chodźki St., PL-20093 Lublin, Poland
[4] Department of General and Coordination Chemistry and Crystallography, Institute of Chemical Sciences, Faculty of Chemistry, Maria Curie-Skłodowska University, M. Curie-Skłodowskiej Sq. 2, PL-20031 Lublin, Poland
* Correspondence: agnieszka.kaczor@umlub.pl, Tel +48 81 448 7273 (A.A.K.)

Supplementary Table S1 and S2.
Supplementary Figure S1- S4.
^{1}H and ^{13}C NMR, HRMS and IR spectra of the investigated compounds.

Table S1. Interatomic distances and selected bond angles for D2AAK5.

Bond lengths (Å)			
C1-C2	1.364(4)	C11-C12	1.500(3)
C1-C8	1.438(4)	C12-C13	1.384(4)
C1-C9	1.501(3)	C12-C20	1.394(4)
C2-N1	1.379(3)	C13-C14	1.387(3)
C3-N1	1.376(4)	C14-C15	1.389(4)
C3-C4	1.398(4)	C14-Cl1	1.732(3)
C3-C8	1.419(3)	C15-O1	1.373(3)
C4-C5	1.360(4)	C15-C19	1.404(4)
C5-F1	1.367(3)	C16-O1	1.450(3)
C5-C6	1.386(4)	C16-C17	1.503(4)
C6-C7	1.382(4)	C17-C18	1.515(4)
C7-C8	1.395(4)	C18-O2	1.449(3)
C9-C10	1.522(4)	C19-O2	1.372(3)
C10-N2	1.495(3)	C19-C20	1.388(3)
C11-N2	1.492(3)		
Bond angles (º)			
C2-C1-C8	107.3(2)	N2-C10-C9	112.0(2)
C1-C2-N1	109.6(3)	N2-C11-C12	113.4(2)
C3-N1-C2	109.1(2)	C11-N2-C10	111.9(2)
N1-C3-C8	107.6(2)	C15-C14-Cl1	119.1(2)
C4-C5-F1	118.1(2)	C13-C14-Cl1	118.9(2)
F1-C5-C6	117.0(3)	C15-O1-C16	116.0(2)
C3-C8-C1	106.42)	C19-O2-C18	117.7(2)
C1-C9-C10	110.6(2)		
Torsion angles (º)			
O1-C16-C17-C18	68.1(3)	C17-C16-O1-C15	-78.9(3)
C16-C17-C18-O2	-66.6(3)	C15-C19-O2-C18	-59.2(4)
O1-C15-C19-O2	-1.2(4)	C17-C18-O2-C19	76.8(3)
C19-C15-O1-C16	60.9(4)		

Table S2. Hydrogen bonding and C-H···*Cg* interactions geometry.

Hydrogen bond [Å, º]				
**D-H···A**	**d(D-H)**	**d(H···A)**	**d(D···A)**	**∠ DHA**
N1-H1N···O1^{i}	0.82(3)	2.26(3)	3.034(3)	157(3)
O3-H1W···Cl2	0.89	2.35	3.209(2)	164
O3-H2W···Cl2^{ii}	0.92	2.33	3.255(2)	176
N2-H2N···Cl2	0.98(3)	2.26(3)	3.217(3)	169(3)
N2-H3N···Cl2^{iii}	0.97(3)	2.17(3)	3.130(3)	175(2)
C2-H2···F1^{iv}	0.95	2.40	3.338(3)	168
C10-H10B···O2^{v}	0.99	2.55	3.424(3)	147
C11-H11B···O3	0.99	2.57	3.337(4)	134
C-H···Cg interactions [Å, º]				
**C-H ···*Cg***	**d(D-H)**	**d(H···Cg)**	**d(C···Cg)**	**∠ CH*Cg***
C16–H16B···*Cg*1^{ii}	0.99	2.43	3.399(3)	165
C16–H16B···*Cg*5^{ii}	0.99	2.78	3.651(3)	147

Symmetry codes: (i) x,y,z-1; (ii) -x+1,-y+1,-z; (iii) -x+1,-y+2,-z; (iv) x,y+1,z; (v) -x+2,-y+2,-z; *Cg*1 and *Cg*5 are the centroids of the N1/C2/C1/C8/C3 and N1/C2/C1/C8/C7/C6/C5/C4/C3 rings, respectively.

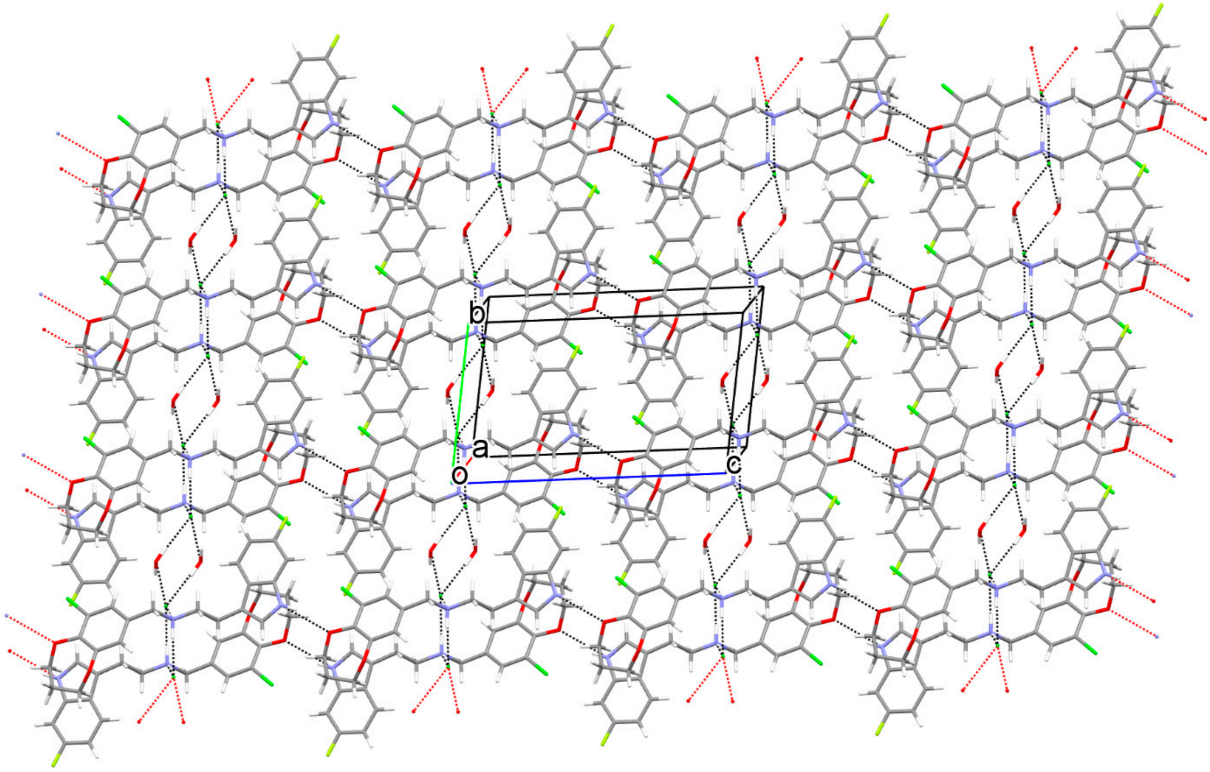

Figure S1. Packing diagrams of D2AAK5 showing formation sheets viewed along the *a*-axis. Hydrogen bonds are indicated by dotted lines.

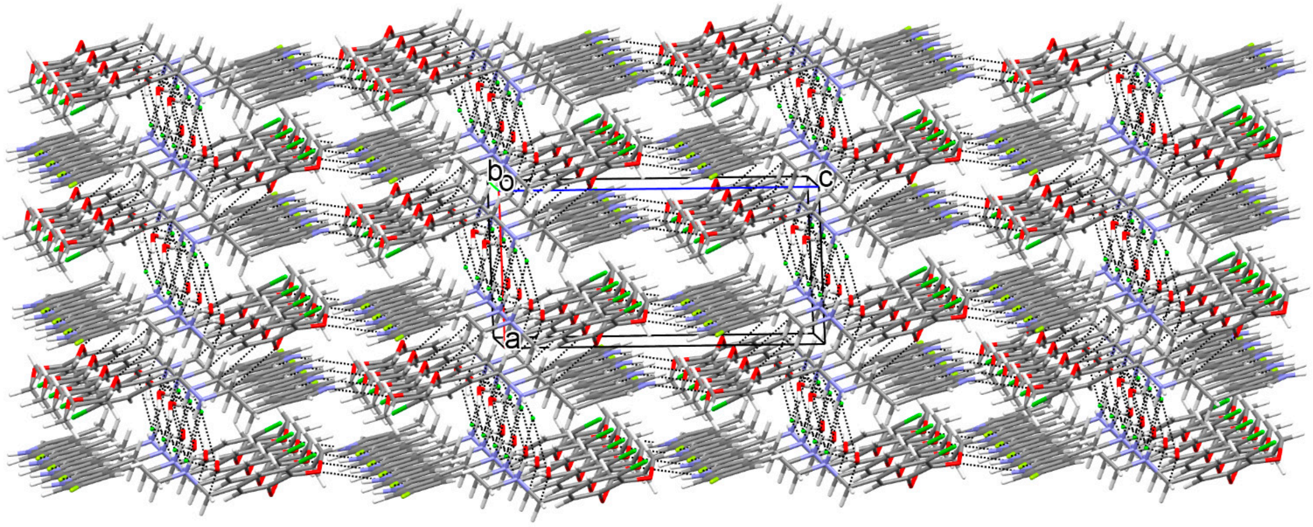

Figure S2. The 3D supramolecular structure of the D2AAK5 constructed by the connection of adjacent sheets through C10–H1B···O2 hydrogen bonds. The hydrogen bonds shown as dotted lines. Hanging contact are omitted for clarity.

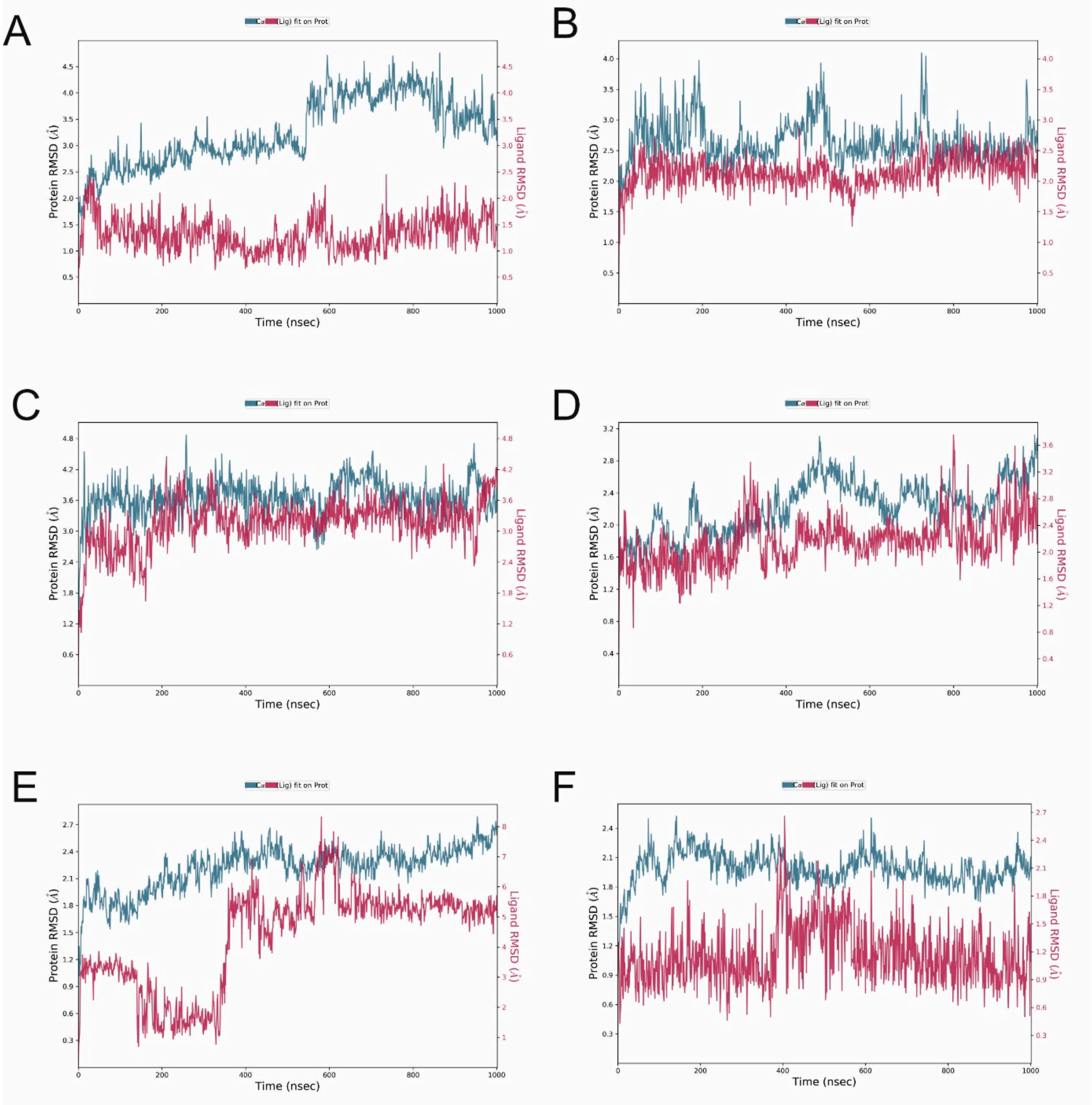

Figure S3. Ligand (purple) and protein (blue) Cα RMSD during 1 µs MD simulations for D2AAK5-5HT$_{1A}$ (A), D2AAK6-5HT$_{1A}$ (B), D2AAK7-5HT$_{1A}$ (C), D2AAK5-5-HT$_{2A}$ (D), D2AAK6-5HT$_{2A}$ (E) and D2AAK7-5HT$_{2A}$ (F) ligand-receptor systems.

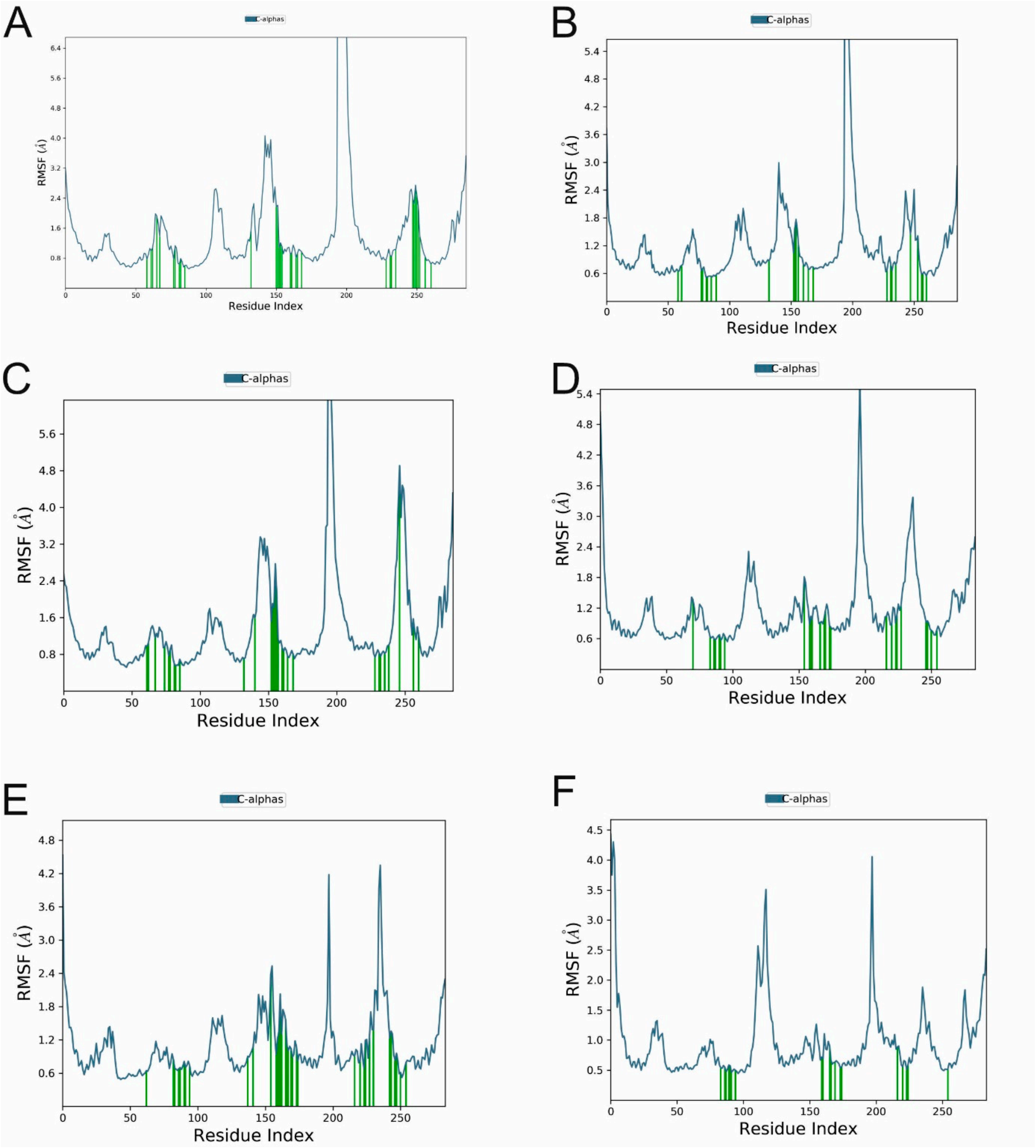

Figure S4. Protein Cα RMSF during 1 μs MD simulations for D2AAK5-5HT1A (A), D2AAK6-5HT1A (B), D2AAK7-5HT1A (C), D2AAK5-5-HT2A (D), D2AAK6-5HT2A (E) and D2AAK7-5HT2A (F) ligand-receptor systems. Protein residues that interact with the ligand are marked with green-colored vertical bars.

N-((9-chloro-3,4-dihydro-2H-benzo[b][1,4]dioxepin-7-yl)methyl)-2-(6-fluoro-1H-indol-3-yl)ethan-1-amine hydrochloride (D2AAK5)

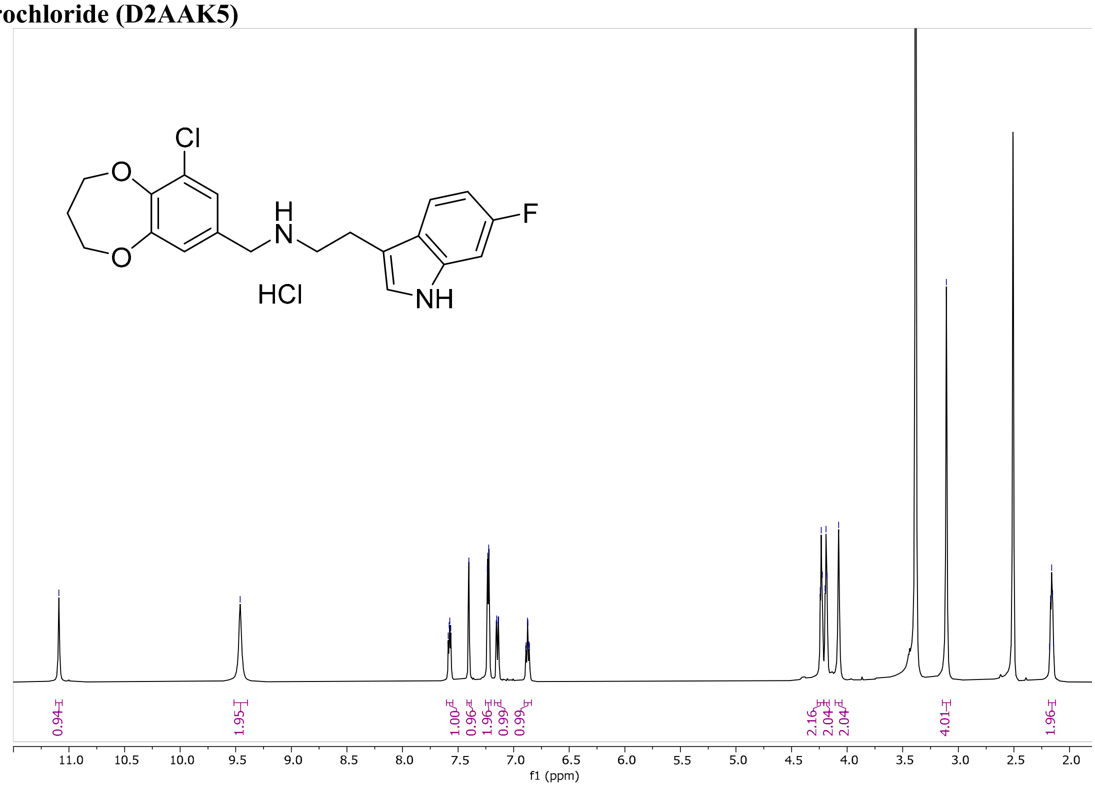

^{1}H NMR (600 MHz, DMSO) δ 11.09 (s, 1H), 9.46 (s, 2H), 7.58 (dd, J = 8.7, 5.3 Hz, 1H), 7.40 (s, 1H), 7.23 (dd, J = 7.3, 2.3 Hz, 2H), 7.15 (dd, J = 10.1, 2.4 Hz, 1H), 6.87 (td, J = 9.2, 2.3 Hz, 1H), 4.23 (t, J = 5.5 Hz, 2H), 4.19 (t, J = 5.4 Hz, 2H), 4.08 (s, 2H), 3.11 (s, 4H), 2.16 (p, J = 5.5 Hz, 2H).

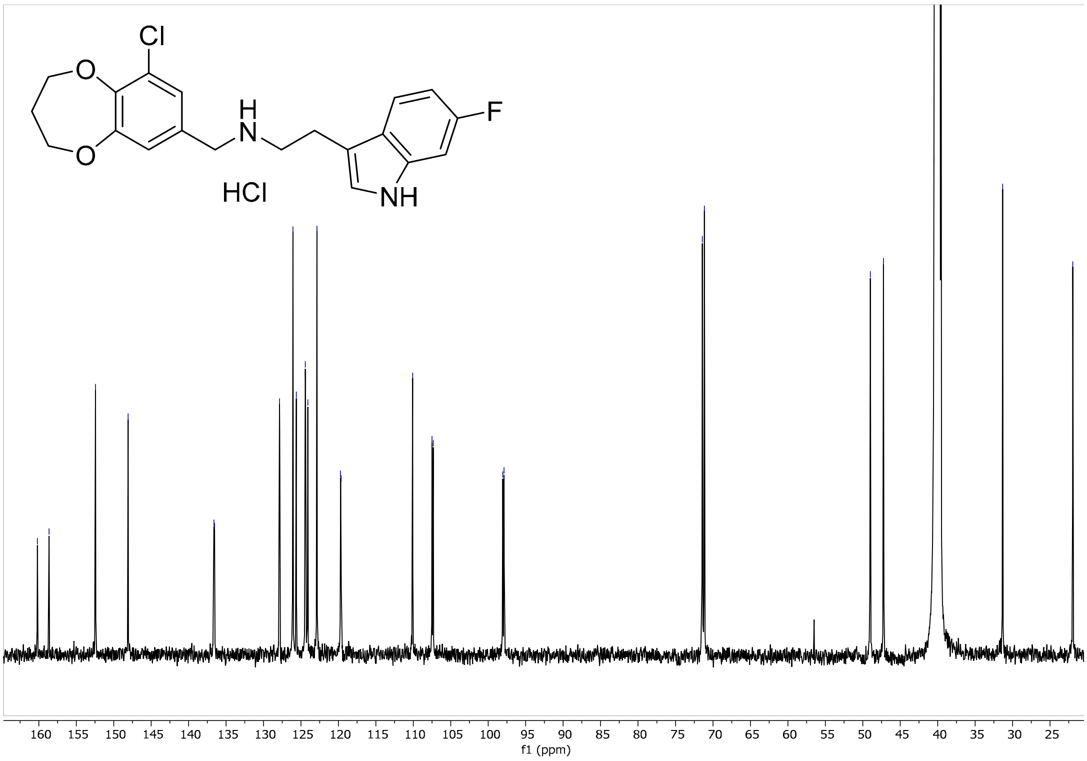

^{13}C NMR (151 MHz, DMSO) δ 159.41 (d, J = 234.2 Hz), 152.43, 148.07, 136.59 (d, J = 12.7 Hz), 127.86, 126.08, 125.62, 124.42, 124.09, 122.86, 119.68 (d, J = 10.2 Hz), 110.09, 107.42 (d, J = 24.4 Hz), 97.97 (d, J = 25.5 Hz), 71.42, 71.15, 49.00, 47.23, 31.33, 21.96.

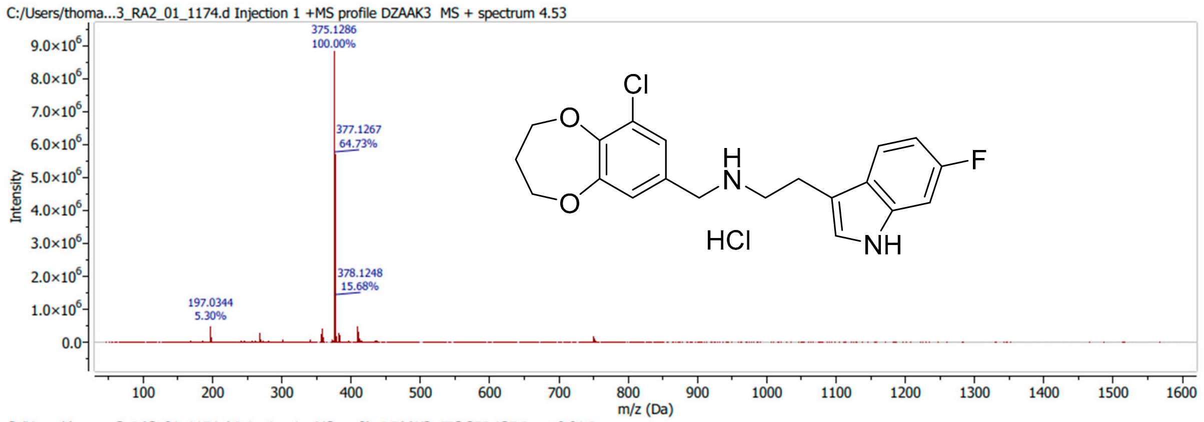

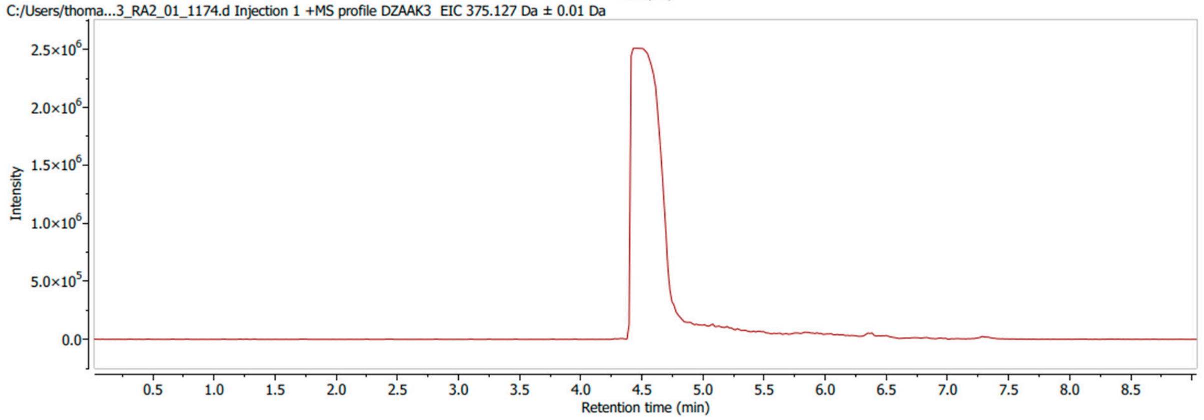

HRMS (ESI) calc. $(M+H)^+$ 375.1270 exp. $(M+H)^+$ 375.1286

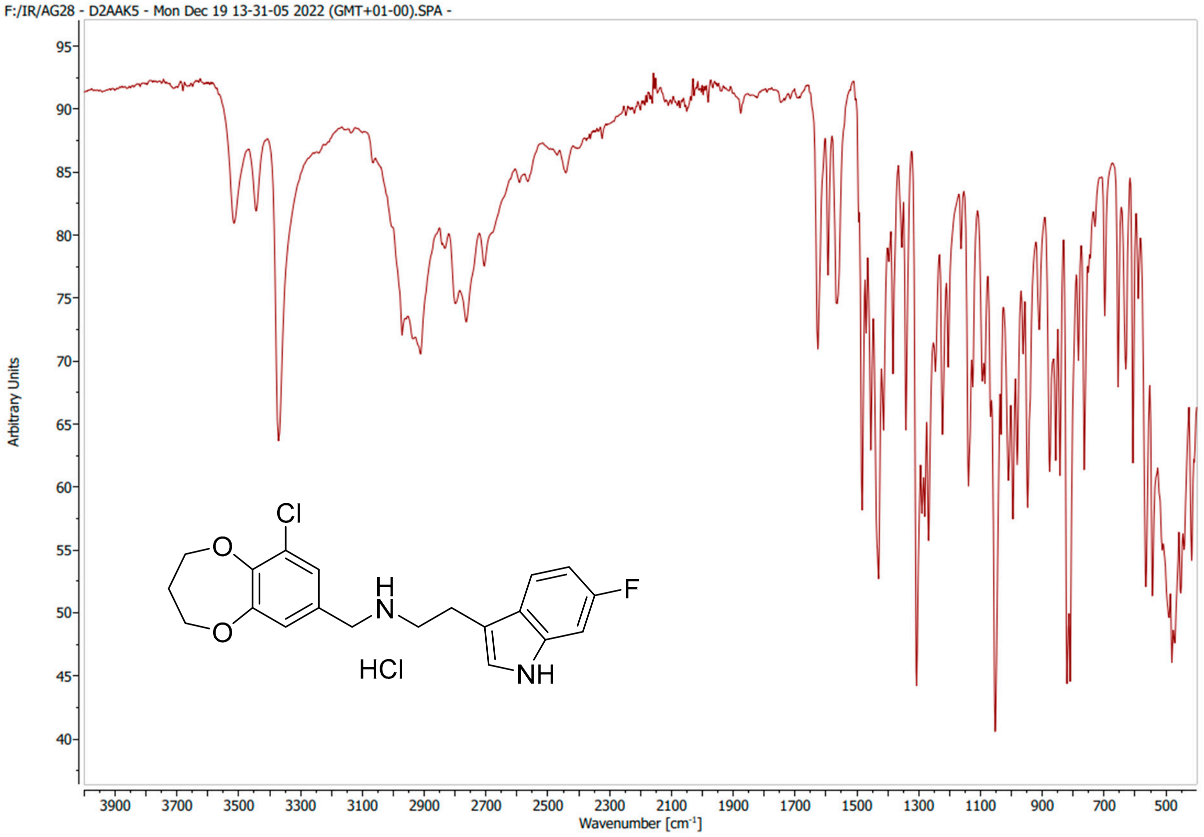

1-((2-(1H-inden-3-yl)ethyl)amino)-3-(4-fluorophenoxy)propan-2-ol (D2AAK6)

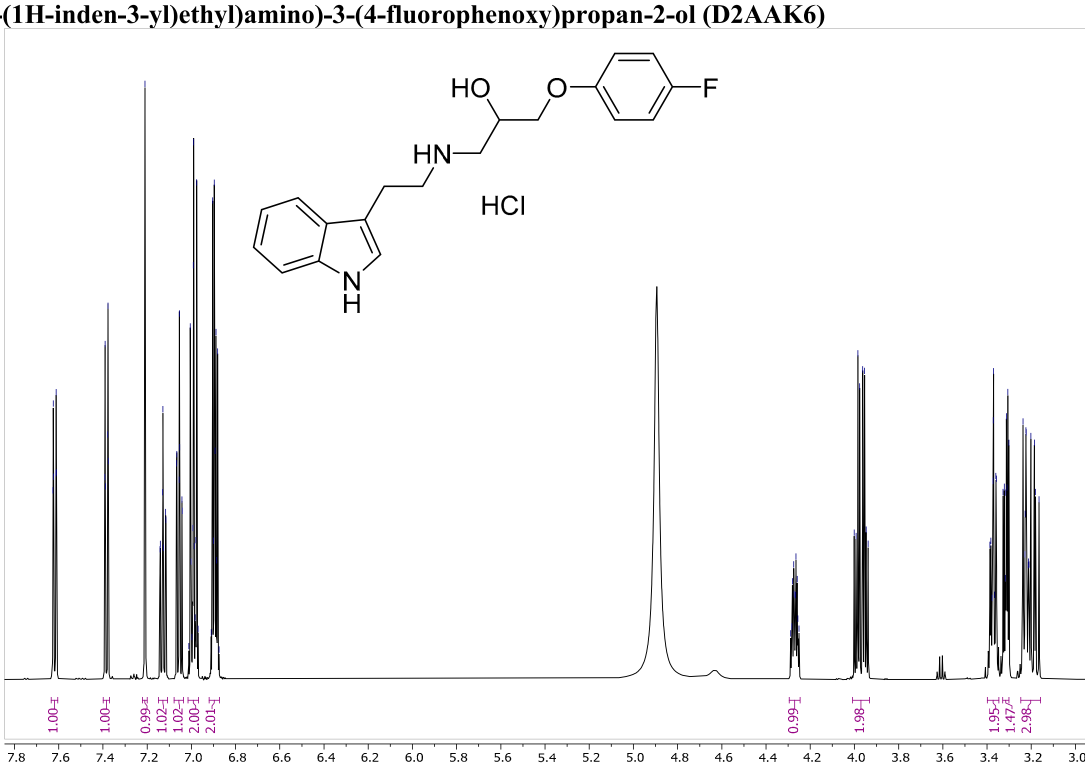

^{1}H NMR (600 MHz, MeOD) δ 7.62 (dt, J = 7.9, 1.0 Hz, 1H), 7.38 (dt, J = 8.2, 0.9 Hz, 1H), 7.21 (s, 1H), 7.13 (ddd, J = 8.1, 7.1, 1.1 Hz, 1H), 7.05 (ddd, J = 8.0, 7.0, 1.0 Hz, 1H), 7.02 – 6.97 (m, 2H), 6.92 – 6.87 (m, 2H), 4.27 (dtd, J = 8.3, 5.1, 3.2 Hz, 1H), 4.01 – 3.93 (m, 2H), 3.40 – 3.35 (m, 2H), 3.33 – 3.30 (m, 1H), 3.25 – 3.16 (m, 3H).

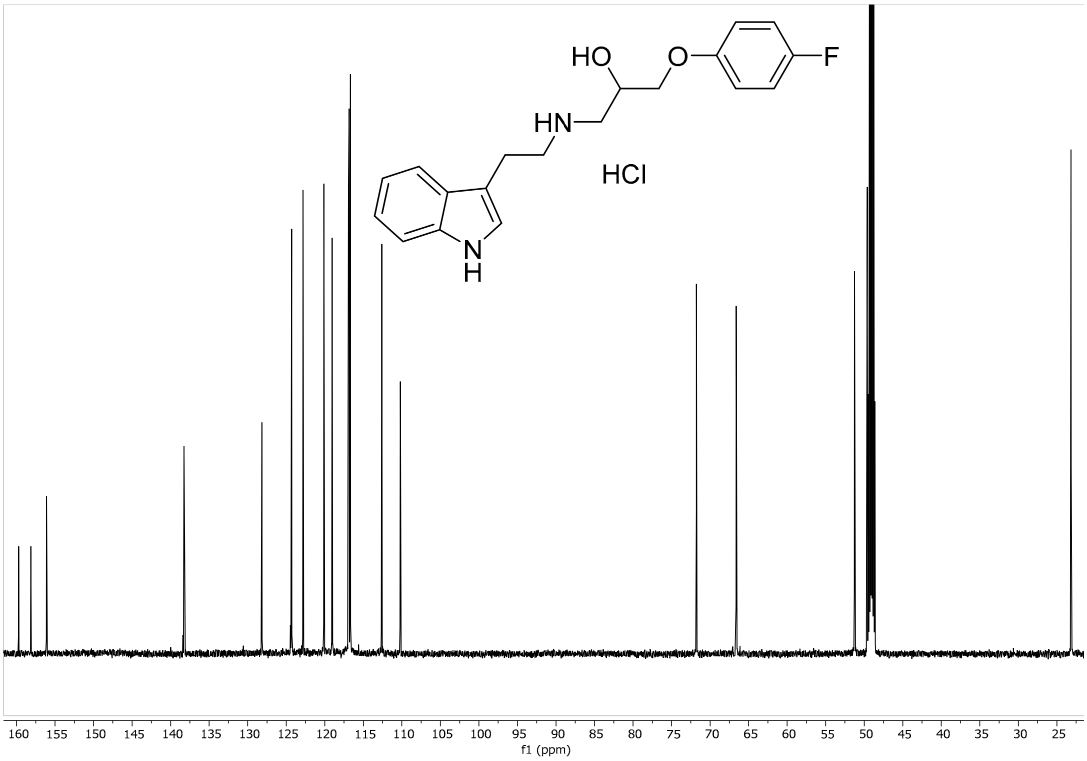

^{13}C NMR (151 MHz, MeOD) δ 159.7, 158.1, 156.1, 156.1, 138.3, 128.2, 124.3, 122.8, 120.1, 119.0, 116.9, 116.9, 116.8, 116.7, 112.6, 110.2, 71.8, 66.6, 51.3, 49.6, 23.2.

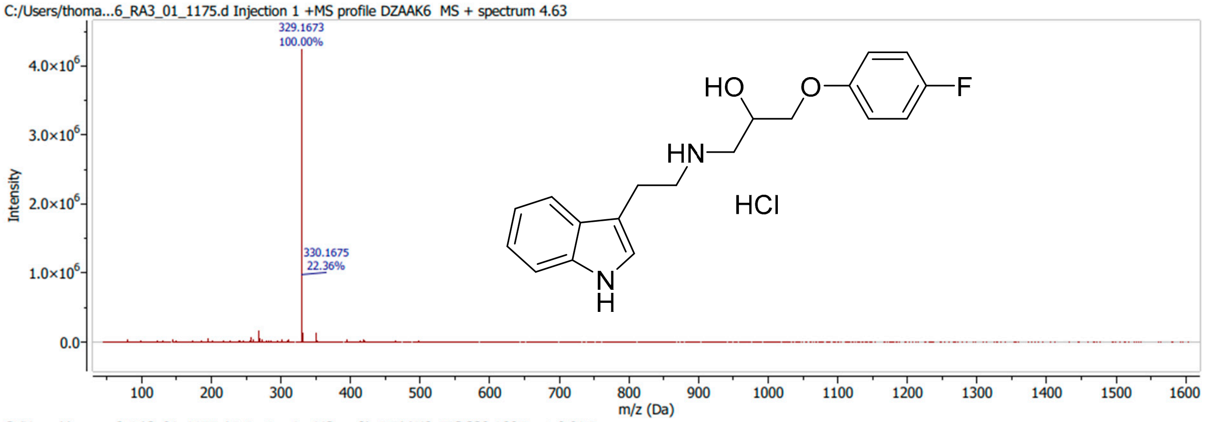

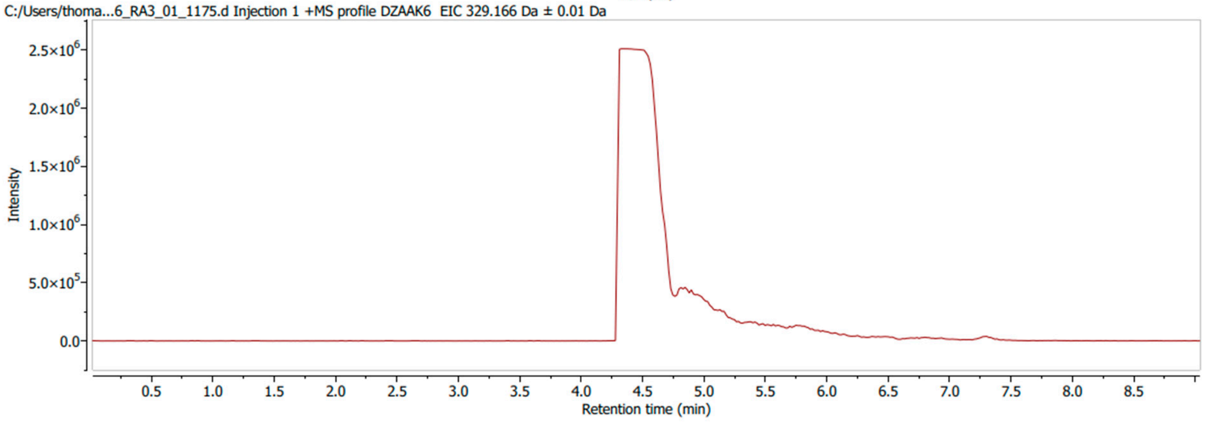

HRMS (ESI) calc. $(M+H)^+$ 329.1660 exp. $(M+H)^+$ 329.1673

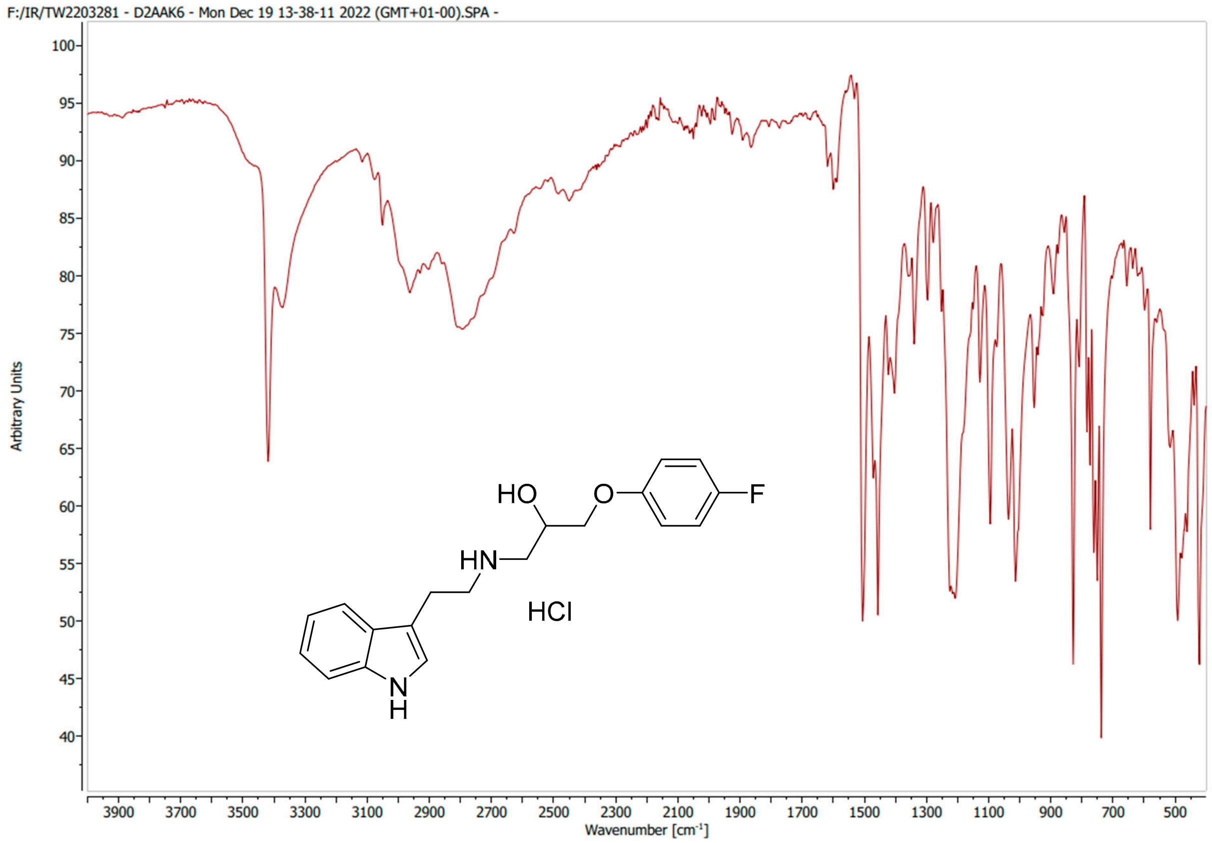

1-((2-(1H-inden-3-yl)ethyl)amino)-3-phenoxypropan-2-ol (D2AAK7)

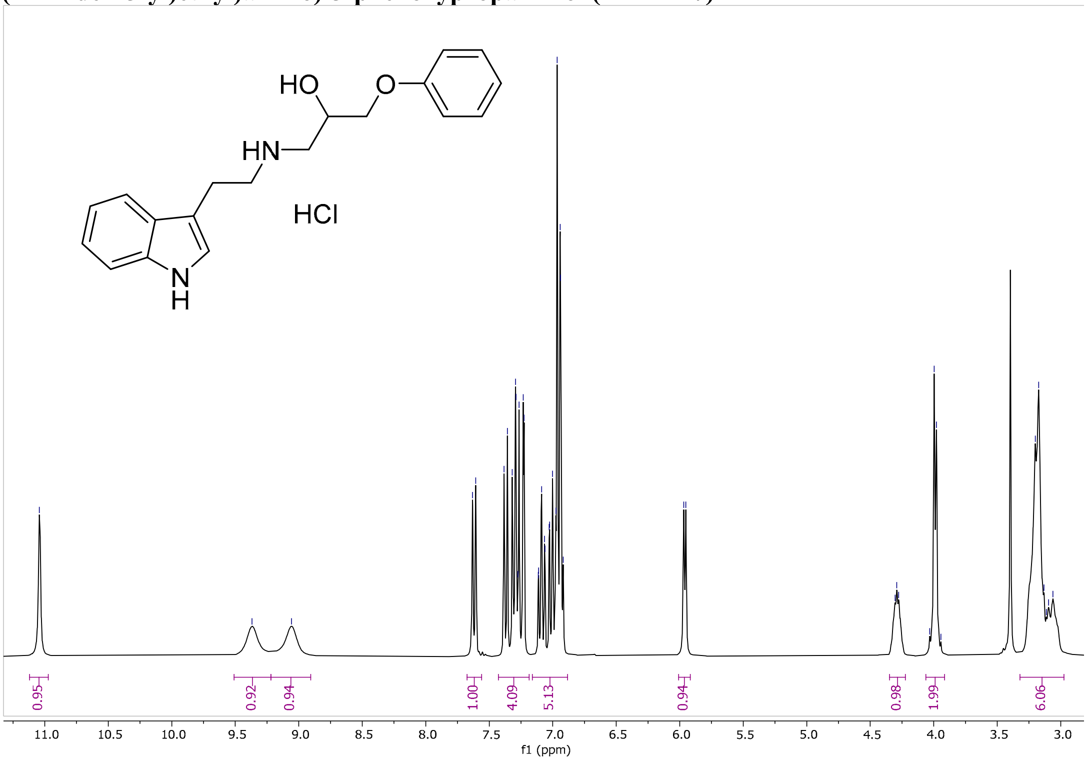

^{1}H NMR (300 MHz, DMSO) δ 11.04 (s, 1H), 9.37 (s, 1H), 9.06 (s, 1H), 7.62 (d, J = 7.7 Hz, 1H), 7.43 – 7.19 (m, 4H), 7.16 – 6.88 (m, 5H), 5.96 (d, J = 4.9 Hz, 1H), 4.35 – 4.22 (m, 1H), 4.06 – 3.91 (m, 2H), 3.32 – 2.97 (m, 6H).

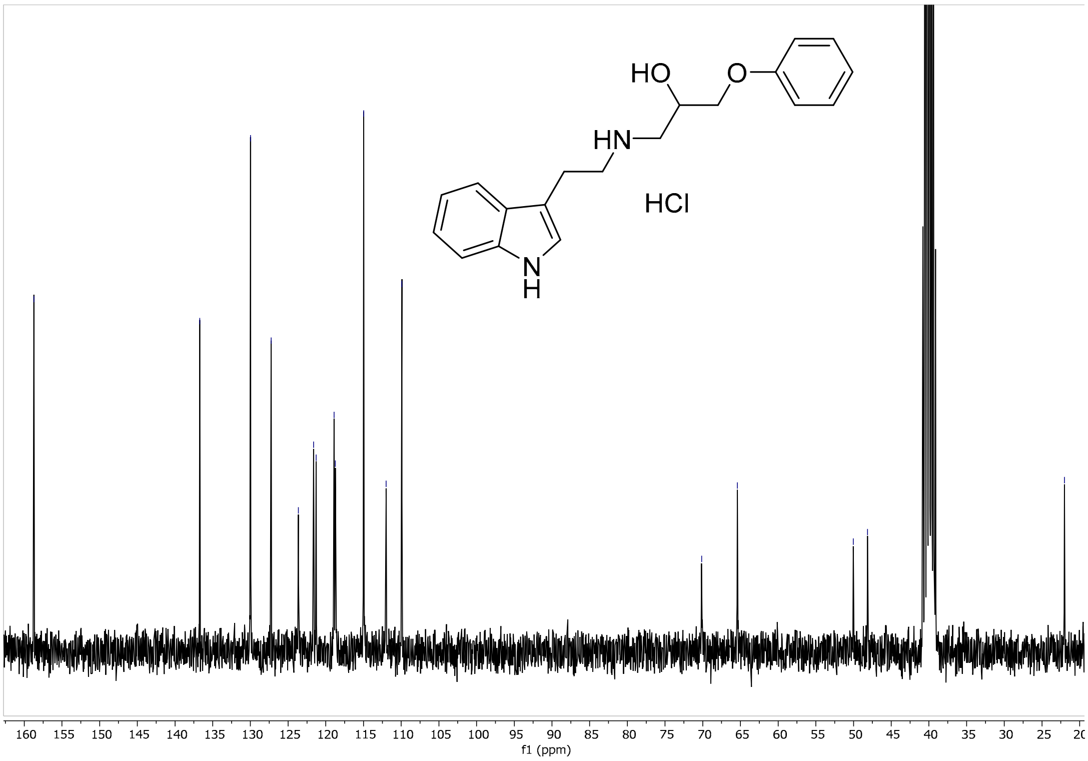

^{13}C NMR (75 MHz, DMSO) δ 158.7, 136.7, 130.0, 127.2, 123.6, 121.6, 121.3, 118.9, 118.7, 115.0, 112.0, 109.9, 70.2, 65.4, 50.0, 48.1, 22.0.

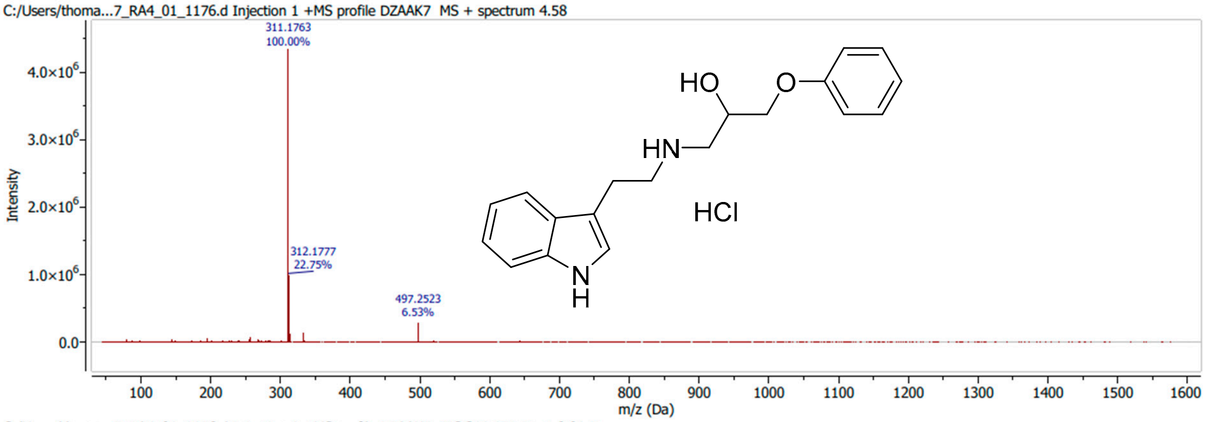

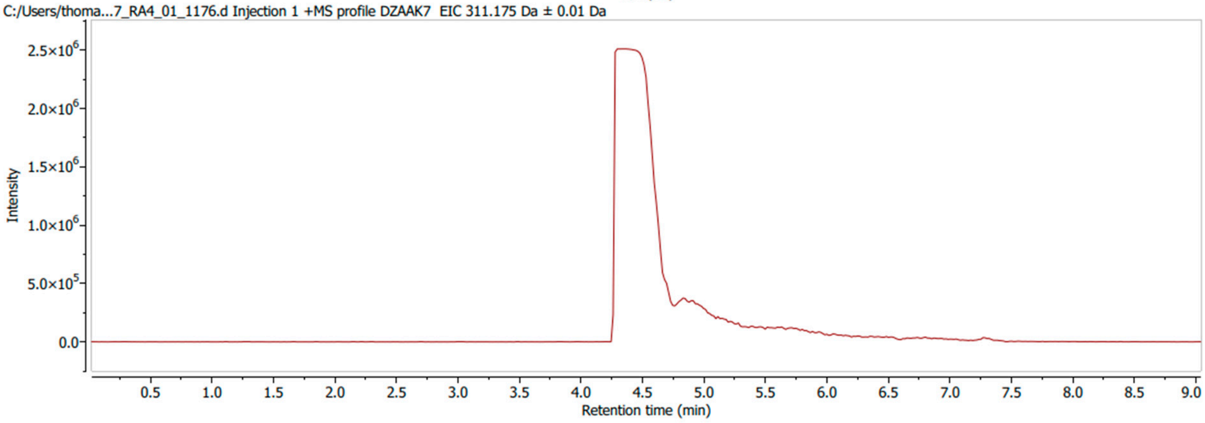

HRMS (ESI) calc. $(M+H)^+$ 311.1754 exp. $(M+H)^+$ 311.1763

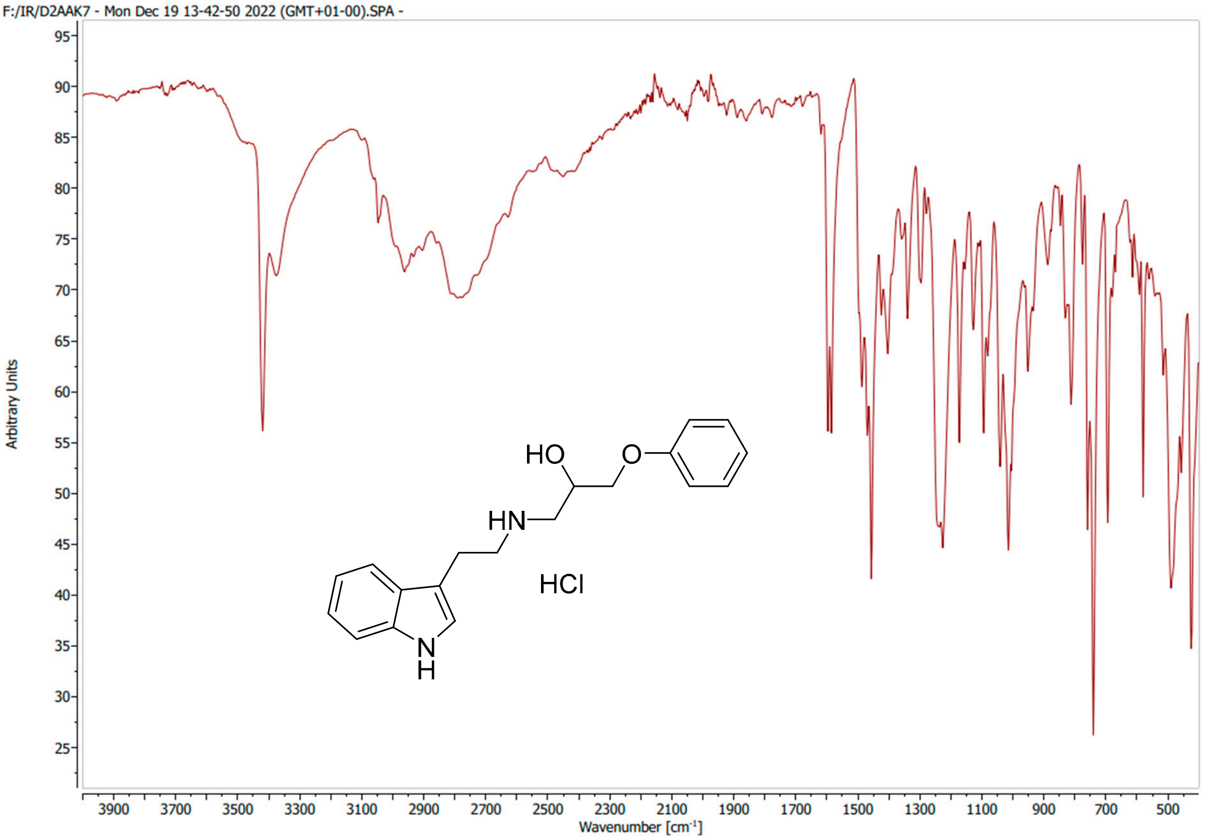

Supplement: Supplementary file 1 [file molecules-28-00383-s001.zip › molecules-2106877-supplementary.pdf]
